# Characterization and Application of Water Kefir-Derived Dextran as Coatings with Chitosan and Antagonistic Yeasts for Strawberry Preservation

**DOI:** 10.1021/acsomega.6c08930

**Published:** 2026-10-14

**Authors:** Bilal Agirman

**Affiliations:** Engineering Faculty, Department of Food Engineering, 37506 Cukurova University, 01330 Adana, Türkiye

## Abstract

This study developed alternative biobased coatings using dextrans extracted from household- and industrial-type water kefir grains (WKG) to extend the postharvest shelf life of perishable strawberries. Extracted dextrans were characterized for their structural, physicochemical, antioxidant, and antifungal properties, and the most promising exopolysaccharide was evaluated as a coating matrix alone or combined with chitosan and antagonistic yeasts under cold (4 °C) and ambient (25 °C) storage. Dextran extracted from household-type grain (D-WKS) exhibited significantly higher antioxidant activity across all three assays than dextran extracted from industrial-type grain (D-WKD) (57.40 vs 49.30% for DPPH, 60.70 vs 51.80% for ABTS, and 63.70 vs 56.60 μmol TE/L for FRAP). Furthermore, D-WKS displayed superior antifungal activity against eight postharvest mold pathogens, demonstrating larger inhibition zones (4.90–23.01 mm for D-WKS vs 1.43–13.54 mm for D-WKD) and lower minimum inhibitory concentration (MIC) values (0.1–100 mg/mL for D-WKS vs 1 to >100 mg/mL for D-WKD). Cold storage proved indispensable for quality preservation. Under cold storage, individual chitosan treatment was effective in limiting weight loss and maintaining fruit pH, °Brix, firmness, and chroma values. However, household-WKG dextran combined with chitosan (D-WKS+Cht) was the most effective in maintaining surface lightness (L), total phenolics, and redness (+a). Notably, after 10 days of storage at 4 °C, D-WKS alone most effectively suppressed disease severity (5.45%), infection ratio (9.09%), and enzymatic browning (ΔBI = 0.089) while achieving the highest sensory preference and consumer purchase intention. Overall, utilizing water kefir-derived dextran alone or in combination with chitosan under cold storage offers a promising and eco-friendly biocontrol strategy for strawberry preservation.

## Graphical abstract

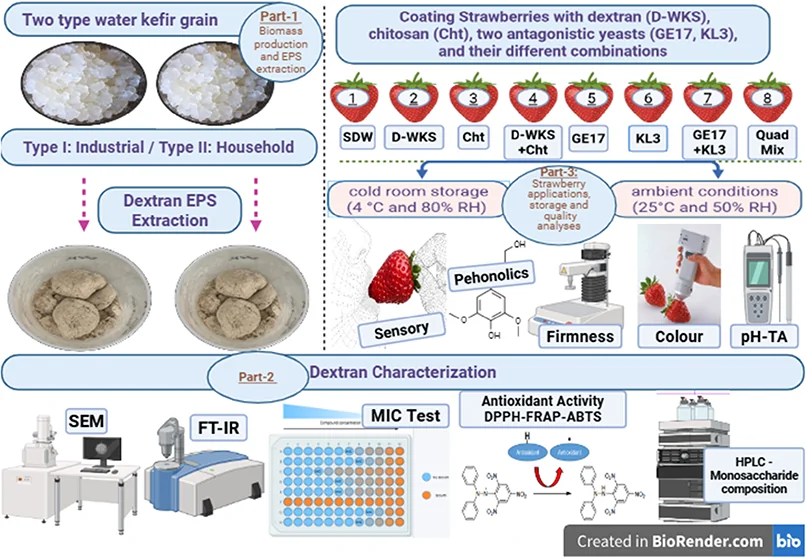

## 1 Introduction

Strawberry (*Fragaria* × *ananassa*) is one of the most popular berry crops worldwide due to its unique flavor, attractive smell, excellent taste, and nutritional attributes. It contains many health-beneficial compounds in abundance, such as nutrients, minerals, antioxidants (polyphenolics, flavonoids, vitamin C, and anthocyanins), folate, potassium, and fiber.^1,2^ Strawberries are an important fruit for Italy, and production and planting amounts are increasing year by year. According to the Food and Agriculture Organization (FAO), Italy was the 13th biggest producer of strawberries in the world in 2024, and the fourth largest in the European Union. The most recent data reported by FAO in 2024 indicates that the yield reached 28,276.3 kg/ha, while the production quantity was equal to 123.85 thousand tons.^3^

One of the most serious issues with strawberries is their short postharvest life (less than 5 days at ambient temperature) due to rapid deterioration. Strawberries have a low mechanical resistance, dehydrate quickly, and are susceptible to fungal infections, making them one of the most sensitive fruits.^4–6^ Among these factors, losses of fruits and vegetables due to fungal infections in the food chain can amount to more than 25% of total production in industrialized countries and more than 50% in developing nations if postharvest handling and storage conditions are inadequate.^7^ *Botrytis cinerea*, which causes gray mold disease, is the most frequent postharvest spoilage fungus that affects strawberries.^8^ Strawberries are typical hosts of this fungus, and it has resulted in substantial commercial losses. The primary signs observed in infected strawberry fruit are pale brown lesions covering the entire fruit surface within 48 h, accompanied by a reduction in fruit firmness. Postharvest, decay triggered by *B. cinerea* leads to a loss ranging from 25 to 35% of strawberry fruit, or approximately USD 10–100 million annually.^2,9^ In addition to economic concerns, infected foods may represent a health risk since some fungi, including *Alternaria*, *Penicillium*, and *Fusarium*, produce mycotoxins, which are secondary metabolites that are detrimental to human health. Therefore, various strategies have been developed to prevent pathogen-induced fruit postharvest loss.^10^ Today, synthetic fungicides represent the first approach for controlling postharvest spoilage in fruits and vegetables worldwide.^11^ However, the emergence of fungicide resistant pathogen biotypes and the increasing awareness of consumers toward the health implications of hazardous chemicals imposed an urgent need of reducing the use of synthetic fungicides in food supply; this phenomenon strengthened the search for alternative biocontrol strategies that are more effective, safer, nontoxic, low residue, environmentally friendly and cost-effective.^12^ Various strategies are used to reduce postharvest spoilage, such as microbial antagonists (yeast, mold, or bacteria),^13^ hydrogen peroxide, citric acid, and ethanol applications,^14^ polysorbate use,^15^ ozone (O_3_) application,^16^ modified atmosphere packaging, ultraviolet-C application,^17^ γ irradiation,^18,19^ biopolymers^20,21^ and the use of bioactive natural ingredients.^22,23^

Among various type of microorganisms, utilization of antagonistic yeasts has emerged as a promising way in order to manage the decay in fruits and vegetables and reduce the use of synthetic fungicides.^24^ Yeasts have gained special attention as postharvest biocontrol agents because of many features which make them particularly suitable as antagonistic agents since they are genetically stable, capable of surviving under adverse environmental conditions (low/high temperature, dry surfaces, oxidative stress, etc.), have simple nutritional requirements, grow rapidly on fruit and vegetables surfaces, colonize in injured areas of fruit and vegetables, could be effective against a wide range of pathogens, are resistant to pesticides, and generally not produce metabolites that are detrimental to human health.^11,25^

Dextran, a polysaccharide that contains predominantly α-(1 → 6) linked glucosyl units, is the main α-glucan in water kefir grains (WKG).^26^ This exopolysaccharide (EPS) is produced extracellularly in a medium high in sucrose as a carbon source by the action of a secreted enzyme known as a dextransucrase, which is produced by some lactic acid bacteria (LAB) species linked to WKG.^27^ Based on the literature, dextran is useful as coatings or biodegradable film-forming agents with antioxidant and antimicrobial characteristics.^27,28^ The use of biobased compounds, such as dextran, in postharvest disease management systems has gained significance in the seek for an alternative to synthetic fungicides. Chitosan, another natural carbohydrate polymer,^29^ is a deacetylated derivative of chitin (the second most abundant renewable biopolymer in nature) and has been commonly used for industrial applications due to its biodegradability, biocompatibility, and nontoxicity characteristics.^30,31^ One of these applications is using chitosan as an edible film coating because of its excellent film properties.^32^ In addition to the durable, flexible, and easily tearable properties of chitosan film, coating fruit and vegetable surfaces with chitosan film can be a useful substitute for common chemical fungicides due to its good antimicrobial activity against various microorganisms, such as Gram-positive and Gram-negative.^33^ Using chitosan film as an edible coating material offers several benefits. For instance, chitosan-based coatings can be employed as a carrier for functional substances such as antioxidants, antimicrobials, minerals, and vitamins. Additionally, edible film coating can act as a barrier, reducing quality losses (particularly weight loss), inhibiting gas exchanges, lowering respiration rate and ethylene generation, and preventing the growth of microorganisms that cause fruit decay.^34,35^ All of these qualities give chitosan an advantage over other polymers. Many studies have examined the use of chitosan as an edible film coating, and it has been reported that it works successfully for a variety of fruits and vegetables, including fresh-cut broccoli,^36^ pear,^37^ pomegranate,^38^ rose apple,^39^ sweet cherry,^40^ mango,^41^ and strawberry.^42^

Many studies investigated the characteristics of biofilms made from various polysaccharides including dextran and chitosan. However, the available studies on dextran-based biofilms have been conducted exclusively *in vitro*, and, to the best of the author’s knowledge, no study has evaluated dextran as a coating material for fruit preservation and postharvest disease management. In addition, as far as it is known, no previous study has investigated the combined application of dextran, chitosan, and antagonistic yeast as a coating system for strawberry preservation. Therefore, the present study aimed to compare the characteristics of industrial and household-type water kefir grains, produce dextran, and evaluate its potential as a strawberry coating material, alone and in combination with chitosan and an antagonistic yeast during storage. The hypothesis of this study was that combining dextran with chitosan and antagonistic yeast would provide greater protection against postharvest deterioration than the individual treatments. This potential complementary effect was hypothesized to arise from the combined film-forming and moisture-retention properties of the biopolymers and the antagonistic activity of the yeast, potentially resulting in enhanced suppression of fungal decay and better maintenance of strawberry quality during storage.

## 2 Materials and Methods

### 2.1 Materials

In this study, two different type (industrial and household) of WKG were utilized. The industrial type was obtained from Danem Inc. (Isparta, Türkiye), while the household type was graciously supplied by a local manufacturer from Sivas, Türkiye. Additionally, the storage studies included chitosan and two different antagonistic yeast species. The chitosan used was purchased from Chem-Lab (CL00.2903.0050, Belgium). Two antagonistic yeasts, *Aureobasidium pullulans* GE17 and *Meyerozyma guilliermondii* KL3, were previously isolated from apple and lemon^11^ and exhibited effective biocontrol capability against some postharvest diseases.^43^ Strawberries (fragole di Marsala) were sourced from local farmers in Marsala, Sicily, Italy.

### 2.2 Biomass Production

WKG were propagated in a specific medium through successive subcultures. Using batch fermentation, the grain biomass was increased to 100 g in a 1 L sterilized glass bottle that had been sterilized. The volume of the fermentation vessel was increased to 3 L when the grain weight exceeded 100 g. WKG were subjected to 30 successive and independent cultivations with one organic Figure (cut into four pieces), four organic raisins, and 7% organic brown sugar in 1 L of sterile potable water as a substrate. Fermentation was conducted at 25 °C for 2 days for each cultivation period. All WKG gathered at the end of each cultivation was used in the subsequent cultivation, and the biomass increasing ratio (BIR) was computed using eq ([Disp-formula eq1]). Following each single cultivation, the WKG was filtered through a sterile sieve and rinsed with sterile distilled water (SDW). Prior to biomass measurement, WKG were dried for 30 min on a sterile sieve to remove excess water from the grains.

1 $$BIR\;\left(\%\right)=\frac{final\;grain\;mass-initial\;grain\;mass}{initial\;grain\;mass}\times 100$$

### 2.3 Dextran Extraction

The EPS was extracted from the gathered WKG from the last cultivation (30th) using the method described by Lucena et al.,^44^ with slight modifications. WKG was mixed with distilled water in a 1:10 (w/v) proportion and heated at 80 °C for 60 min while vigorously stirring (700 rpm). The mixture was then cooled, and a precipitate was obtained by centrifugation at 6500*g* for 10 min at 4 °C. The precipitate was mixed with a 0.1 N NaOH solution at a ratio of 1:2 (precipitate: NaOH, w: (v) and vigorously stirred (700 rpm) at 80 °C until it was completely dissolved (approximately 60 min). After dissolving in NaOH, the mixture was cooled, filtered through a stainless-steel strainer, and titrated with 0.1 N HCl until it reaches pH 7 for neutralization. After adding absolute cold ethanol (for EPS precipitation) to the neutralized mixture in a 1:3 (v/v, mixture/ethanol) ratio, it was kept overnight at −20 °C. Subsequently, the mixture was centrifuged at 6500*g* for 10 min at 4 °C to obtain the material. The solubilization and precipitation steps were performed twice. Finally, the precipitate was lyophilized, and the resulting pure brown solid polymer was termed “dextran.” Equation 2 was used to compute the yield of dextran extraction.


2 Y=(Z/X)×100×100(Y:Yield(%),Z:Theweightofthelyophilizedsample,X:Theinitialweightofkefirgrains

### 2.4 Characterization of Dextrans

#### 2.4.1 Scanning Electron Microscopy (SEM) Analysis

Scanning Electron Microscopy (SEM-FEI QUANTA 200F) was used to examine the morphology (both external and internal) and structural form of dextran extracts, as described by Lopresti et al.^45^ The microbial consortium and microstructure of freeze-dried WKG were also investigated separately. The samples (3 mm diameter) were attached to an aluminum stub using adhesive carbon tape. To eliminate electrostatic discharge during the test, they were sputter coated with gold (Sputtering Scancoat Six, Edwards) for 60 s under an argon atmosphere prior to imaging. Images were captured at magnifications of 250×, 500×, 1000×, 2000×, 4000×, 8000×, and 16,000× for WKG and 2500×, 5000×, and 10,000× for dextran samples, respectively.

#### 2.4.2 Antioxidant Activity (AA) Assays

This analysis was conducted using three different methodologies, as described below, in order to minimize inconsistent findings and address concerns regarding the reliability of existing in vitro assays expressed by several researchers.^46^ All assays were performed in triplicate (*n* = 3), and the data are reported as mean ± standard deviation (SD).

##### 2.4.2.1 2,2-Diphenyl-1-picrylhydrazyl Radical Scavenging Activity (DPPH)

The ability of the EPS material to scavenge DPPH free radicals was determined using the method described by Vieira et al.,^47^ with slight changes. During testing, 0.5 mL of a 6.0 × 10^–5^ M DPPH (Sigma-Aldrich, D9132, St. Louis, USA) solution in ethanol was mixed with 3.0 mL of aqueous EPS (1 mg/mL). The resulting DPPH and polysaccharide mixture was incubated for 30 min at 25 °C before being measured for absorbance at 517 nm. The inhibition capacity of the dextran extracts in scavenging DPPH radicals was determined in percentage units (I%) using eq ([Disp-formula eq3]).

3 $$I\%=\left[\left(A_{\mathrm{blank}}-A_{\mathrm{sample}}\right)/A_{blank}\;\right]\times 100$$

­(Here *A*_sample_ and *A*_blank_ are the absorbance of a reaction mixture with the polysaccharide solution and a mixture with distilled water in place of the polysaccharide, respectively)

##### 2.4.2.2 2,2′-azino-bis (3-Ethylbenzothiazoline-6-sulfonic acid) Radical Scavenging Activity (ABTS)

The ABTS assay was performed according to a method previously published by Tai et al.,^48^ with minor changes. For each experiment, a fresh ABTS+ (Sigma-Aldrich, A1888, St. Louis, USA) solution was prepared using the same method as Tai et al.^48^ A 3.0 mL sample of the ABTS+ solution was combined with 0.5 mL of an EPS sample solution (1 mg/mL). After 30 min of incubation at 25 °C, the EPS’s scavenging activity in terms of inhibiting the ABTS radical cation was determined using eq ([Disp-formula eq3]) in the same way as the DPPH tests, but with an absorbance at 734 nm.

##### 2.4.2.3 Ferric Reducing Antioxidant Power Assay (FRAP)

The FRAP assay was carried out using a method reported by Liu et al.^25^ Standard or sample solutions with a concentration of 1 mg/mL (0.3 mL) were mixed with 2.7 mL of 2,4,6-tris­(2-pyridyl)-s-triazine reagent (TPTZ, Supelco, 93285, Darmstadt, Germany), which was freshly prepared by mixing a 300 mM acetate buffer at pH 3.6, 10 mM TPTZ in 40 mM HCl, and 20 mM FeCl_3_ in a 10:1:1 volume ratio. The mixtures were subsequently heated at 37 °C for 10 min prior to measuring their absorbance at 593 nm. The results were expressed as μmol Trolox equivalents per liter (μmol TE/L), using Trolox (Sigma-Aldrich, 238813, St. Louis, USA) as the reference standard.

#### 2.4.3 Color Analysis

The color characteristics of the dextran samples were measured using a digital chroma-meter (CR-400, Konica Minolta Inc., Japan). The Hunter color characteristics, including lightness (*L*), redness-greenness (a), and yellowness-blueness (b), were recorded using a standard white calibration plate (*Y* = 93.7, *x* = 0.3135, *y* = 0.3194) as a background for color assessment of EPS.^49^ Additionally, the mean values of three basic chromatic coordinates were utilized to calculate the Chroma (C*_ab_), Hue (*H*°), Whiteness Index (WI), and Total Color Difference (Δ*E*).^50^ The C*_ab_, *H*°, WI and Δ*E* values were calculated using eqs 4, 5, 6 and 7, respectively.

4 $${C^{*}}_{\mathrm{ab}}=\sqrt{{\left(a\right)}^{2}+{\left(b\right)}^{2}}$$

5 $$H^{{}^{\circ}}=\tan^{-1}\frac{\left(b\right)}{\left(a\right)}$$

6 $$\mathrm{WI}=100-\sqrt{{\left(100-L\right)}^{2}+{\left(a\right)}^{2}+{\left(b\right)}^{2}}$$

$$\Delta E=\sqrt{{\left(L^{*}-L\right)}^{2}+{\left(a^{*}-a\right)}^{2}+{\left(b^{*}-b\right)}^{2}}$$ 7

where L*, a*, and b* represent the color coordinates of the instrument’s standard white reference, while *L*, *a*, and *b* represent the corresponding color coordinates measured on the strawberry fruit.

#### 2.4.4 Moisture Content

The moisture content (MC) of the dextrans was determined by measuring the weight loss of extracts before and after drying in a laboratory oven at 105 ± 1 °C until constant weight was reached (dry sample weight).^49^ Three replications of each EPS were used for calculating the MC.

#### 2.4.5 Fourier Transform-Infrared Spectroscopy (FTIR)

The ATR/FT-IR spectra of the lyophilized polysaccharide extracts were analyzed using an FTIR Spectrophotometer (Shimadzu, IRTracer-100, Italy) in accordance with the method provided by Chen et al.^51^ to assess the presence of various functional groups in dextran-EPS. Fourier transform-infrared spectra were recorded at a wavelength of 4000–400 cm^–1^ and a resolution of 4 cm^–1^. For each material, five samples were tested and representative spectra were reported.

#### 2.4.6 Antifungal Efficacy of EPS

The antifungal properties of dextran extracted from industrial and household grains were assessed using two methods as outlined below.

##### 2.4.6.1 Disc Diffusion Test

To select the extracts that inhibit mold growth, the antifungal activities of the dextran extracts were tested against eight molds (details in Table 1) using the disc diffusion method with minor modifications.^50^ All fungi used in this experiment were supplied from the Pathogen Laboratory at the University of Palermo’s (UNIPA) SAAF Department.

**Table 1:** Details on the Molds Used to Test for Antifungal Activity^a^

GenBank accession number	strain name	isolation source
KU729126	*Aspergillus fumigatus* (ATCC-204305)	sputum
Type Strain	*Aspergillus flavus* (BCCM/MUCL18903)	indigenous food
Type Strain	*Aspergillus brasiliensis* (BCCM/MUCL29039)	blueberry
KC692222	*Penicillium italicum* (ML332)	multifloral honey
KC692224	*Penicillium echinulatum* (ML291)	citrus honey
KC692221	*Alternaria alternata* (ML356)	multifloral honey
Type Strain	*Penicillium digitatum* (DSM2750)	molded orange
Type Strain	*Penicillium expansum* (DSM62841)	rotting european crab apple

a ATCC: The American Type Culture Collection, BCCM: Belgian Coordinated Collections of Microorganisms, MUCL: Mycological collection of Université catholique de Louvain (UCLouvain).

First, molds were grown in potato dextrose agar (PDA) medium at 25 °C for 5 days. Suspensions of fungal spores were counted with a hemocytometer (Bürker, Assistant, Germany) and standardized to 10^7^ spores/mL. Subsequently, 100 μL of the prepared spore suspension aliquot was inoculated on the surface of the PDA plate. Then, sterile filter paper disks with a diameter of 5 mm (Whatman No. 1) were stained with 10 μL of dextran extracts with a concentration of 100 mg/mL. The discs were allowed to air-dry before being placed on PDA plates inoculated with fungal pathogens. Each culture was tested in triplicate. Each plate also included one disc containing cycloheximide (1 mg/mL in SDW) (Sigma-Aldrich, C7698, St. Louis, USA) as a positive control and another disc containing SDW as a negative control. The plates were incubated at 25 °C for 72 h. Finally, the diameter of any zones of inhibition present was measured using a digital caliper.

##### 2.4.6.2 Minimum Inhibitory Concentration (MIC) Test

The MIC values were determined using the broth microdilution method in sterile 96-well microplates (0.38 mL capacity, Greiner M3061), following standard protocol guidelines with slight modifications.^52^ Dilution series of freeze-dried dextran were prepared in Potato Dextrose Broth (PDB) at concentrations ranging from 0.001 to 100 mg/mL (100, 10, 1, 10^–1^, 10^–2^, and 10^–3^ mg/mL). Each well contained 100 μL of dextran solution and 100 μL of standardized fungal suspension (10^7^ spores/mL in PDB) (OD_sample_). An untreated fungal growth control containing 200 μL of standardized fungal suspension in PDB (OD_control_), a positive control containing 100 μL of standardized fungal suspension and 100 μL of cycloheximide (1 mg/mL), dextran blanks containing 100 μL of each dextran concentration and 100 μL of PDB without fungal inoculation (OD_blank_), and an uninoculated PDB blank containing 200 μL of sterile PDB (OD_control blank_) were included in each plate. Following 72 h of incubation at 25 °C,^53^ fungal growth was assessed both visually based on visible mycelial development and spectrophotometrically by measuring absorbance at 600 nm using a microplate reader (ScanReady, Life Real P-800). The percentage of fungal growth inhibition was calculated as follows:

$$g\mathrm{rowth}\;\mathrm{inhibition}\;\left(\%\right)=[1-\left(\mathrm{OD}{\;}_{\mathrm{sample}}-\mathrm{OD}{\;}_{\mathrm{blank}}\right)/\left(\mathrm{OD}{\;}_{\mathrm{control}}-\mathrm{OD}{\;}_{\mathrm{control}\;\mathrm{blank}}\right)]\times 100$$

where OD_sample_ represents the absorbance of the dextran-treated fungal culture, OD_blank_ represents the absorbance of the corresponding dextran concentration without fungal inoculation, OD_control_ represents the absorbance of the untreated fungal growth control containing the standardized fungal suspension in PDB, and OD_control blank_ represents the background absorbance of uninoculated PDB. The MIC end point was defined as the lowest dextran concentration resulting in ≥80% inhibition of fungal growth relative to the untreated growth control. Each concentration was tested in four replicates to assess the reproducibility of the MIC measurements.

#### 2.4.7 Monosaccharide Composition of EPS

The monosaccharide composition of dextran samples was measured using a method described by Edis et al.,^54^ with some modifications. Glucose, fructose, and galactose were analyzed using an HPLC (Shimadzu, LC-20AT, Japan) system equipped with a refractive index detector (RID-10A). To determine the monosaccharide composition, a solution of lyophilized EPS samples was prepared. Then, 5 mL of this solution was combined with 15 mL of 1% HCl, and hydrolysis was performed at 90 °C for 3 h. Before injection, hydrolyzed samples were filtered using a 0.45 μm Millipore filter. Then, 20 μL of the supernatant was injected into the Aminex HPX-87H column (300 × 7.8 mm, Bio-Rad, Hercules, CA). The column was maintained at 35 °C, with 3 mM H_2_SO_4_ + 7% acetonitrile as the mobile phase at a flow rate of 0.5 mL/min. Individual sugars were identified by comparing their retention times to those of corresponding commercial standards and quantified using external standard calibration curves.

### 2.5 Strawberry Treatments

Once the characterization was completed, the strawberry fruits were coated with the EPS material that showed the highest antifungal activity. The studies were conducted using 48 kg of strawberries (fragole di Marsala), which were harvested in Marsala, Sicily, and delivered to the laboratory on the same day. First, strawberries were selected and sorted by size, and those that were decaying, wounded, spoilt, or on the verge of deterioration were removed. Intact fruits were first washed with tap water to remove dust, leaves, and other foreign substances, then disinfected with 1% (w/v) sodium hypochlorite (NaOCl) solution for 1 min, rinsed with SDW, and then allowed to dry at room temperature. In addition to EPS, solutions comprising chitosan and two antagonistic yeasts were used to coat strawberries and included in storage experiments. A total of seven different solutions used as coating agents are as follows: water kefir dextran (WKD), chitosan (Cht), WKD + Cht (1:1), *A. pullulans* GE17, *M. guilliermondii* KL3, GE17 + KL3 (1:1), and WKD + Cht + GE17 + KL3 (1:1:1:1). SDW was used as a control treatment. The concentration of chitosan and dextran to be used in the storage study was determined to be 1.5% based on preliminary trials (data not shown). The concentration of yeast solutions used in the experiments was adjusted to 10^8^ cells/ml for each yeast. The above-mentioned dextran, chitosan, and yeast solutions were prepared, and a thin protective layer was formed by immersing the predicted number of strawberries for each experiment in the coating solutions for 2 min. The strawberries were then dried at room temperature for 1 h, and the exact number of strawberries required for each experiment was calculated and packed separately in PET clamshell containers (four packages per treatment). Two independent sets were prepared and stored separately for each treatment, resulting in a total of eight packages per treatment and two biological replicates (*n* = 2).

Strawberries were stored under two conditions: ambient conditions (25 ± 2 °C and 50 ± 5% RH) for 5 days and cold storage (4 ± 2 °C and 80 ± 5% RH)^55^ for 10 days. The different storage durations were selected based on the distinct rates of postharvest deterioration under the two temperature conditions. Strawberries are highly perishable fruits that undergo rapid softening, structural deterioration, and tissue liquefaction at room temperature. Preliminary observations indicated that extending storage at 25 °C beyond 5 days resulted in severe deterioration and loss of structural integrity, making reliable quality assessments difficult. Therefore, 5 days was adopted as a practical storage duration for ambient conditions, whereas 10 days of cold storage allowed the extended protective efficacy of the coating treatments to be evaluated under refrigerated conditions. The Day 10 measurements were therefore used to assess the longer-term performance of the treatments under refrigerated storage rather than for direct comparison with the ambient condition.

All analyses described below were conducted at the beginning of storage (Day 0) and after 5 days of storage under both conditions. For refrigerated storage, the analyses were additionally performed after 10 days of storage.

#### 2.5.1 Weight Loss (WL)

For both the ambient and cold storage strawberry studies, ten strawberries were chosen for each treatment (10 fruits × 8 treatments × 2 storage conditions = a total of 160 strawberry fruits) and assigned corresponding codes. Then, the mass (g) of each fruit was measured twice as technical replicates (*n* = 2) using a balance (±0.0001 g) (Orma, Model BC, Italy) before treatment (*A*) and on each test day (B) during storage. The WL was calculated according to the formula (*A*–*B*)/*A* as a percentage at the end of the storage period.

#### 2.5.2 pH

Strawberry fruit pulp was obtained using a kitchen-type blender (Vestel, Mix-Go), and the pH of the samples was measured in three technical replicates (*n* = 3) directly with a digital pH meter (XS, pH 8 + DHS, Italy).

#### 2.5.3 Titratable Acidity (TA)

To determine TA, 20 mL of fruit pulp from each treatment was placed in a 250 mL beaker, and 50 mL of distilled water was added. The solution was titrated against standardized 0.1 N NaOH until the phenolphthalein end point (pH 8.2 ± 0.1). The analysis was performed in three technical replicates (*n* = 3). The volume of NaOH was converted to grams of citric acid per 100 mL of strawberry pulp (%TA) based on the method of Tomadoni et al.^56^

### 2.5.4 Total Soluble Solids (**°**Brix)

°Brix content was measured using a digital refractometer (DBR 35, Italy). A drop of fruit juice was placed on the refractometer’s panel, and soluble solids were measured in three technical replicates (*n* = 3).

#### 2.5.5 Firmness

Fruit firmness was evaluated at two opposite sides of each individual fruit by a digital penetrometer (Model 53205, TR Turoni & C. snc., Forlì, Italy) fitted with a cylindrical stainless steel probe of 5 mm diameter. Firmness was calculated as the mean of four measurements (two measurements per side; *n* = 4 technical replicates) along the equatorial zone of each strawberry, and the results were expressed in Newton (N).^57^ On each sampling day, four fruits from each treatment were used for measurement.

#### 2.5.6 Color Analysis

The external skin color of strawberry fruits was measured using a chroma-meter. When the trials were first set up, five strawberry fruits for each treatment (8 treatments × 2 storage conditions × 5 fruits = 80 strawberries) were placed separately in alcohol-sterilized PET clamshell packages for use only in color analysis, thereby standardizing the packaging conditions and minimizing potential environmental effects on color measurements. The color parameters *L*, *a*, *b*, C*_ab_, *H*°, and Δ*E* were measured on both sides of each strawberry fruit during storage, with three technical measurements performed on each side (n = 3 per side), using the method described in section 2.4.3, and mean values were computed.

#### 2.5.7 Browning Index (BI)

The BI was determined using the method described by Aguiló-Aguayo et al.,^58^ with several modifications. Ten mL of freshly extracted fruit pulp were mixed with 10 mL of distilled water and were centrifuged at 14.000 rpm for 10 min at 4 °C. Then supernatants were then mixed with 95% ethanol at a ratio of 1:1 and transferred into new falcon tubes. The mixture was vortexed vigorously and centrifuged again at 14.000 rpm for 10 min at 4 °C. Finally, the absorbance of the supernatants was recorded in three technical replicates (*n* = 3) at 420 nm against a blank (95% ethanol) using a UV–vis spectrophotometer (Jenway 6400 Visible-spectrophotometer, UK).

#### 2.5.8 Total Phenolic Content (TPC)

The TPC of the samples was determined using the Folin-Ciocalteu method described by Sarıdaş et al.^59^ Briefly, 100 μL of extract was mixed with 100 μL Folin-Ciocalteu’s phenol reagent (Merck, 109001, Darmstadt, Germany) and 3000 μL ultrapure water in glass tubes. After 10 min of incubation, 100 μL of Na_2_CO_3_ solution (20%, w/v) was added to the tubes. The mixture was then incubated in darkness for 2 h to achieve reaction equilibrium. The absorbance was measured in two technical replicates (*n* = 2) at 765 nm using a spectrophotometer (Jenway 6400 Visible Spectrophotometer). Gallic acid was used as a standard, and TPC was expressed as milligrams of gallic acid equivalents per 100 g of sample (mg GAE/100 g).

#### 2.5.9 Disease Severity (DS) and Infection Ratio (IR)

Eleven strawberries were used for each treatment in each of the storage conditions. Experiments were carried out in duplicate, with a total of 352 strawberries employed (2 storage conditions × 8 treatments × 11 fruits × 2 parallel set-ups = 352). IR, also known as decay incidence (DI), was visually examined daily during the storage period. Strawberries with surface mycelia growth, microbial lesions, or rot are regarded as decayed.^60^ The IR for each treatment was determined using eq 8 and expressed as a percentage of infected fruits.^8^

8 IR(%)=(numberofinfectedfruit/totalfruit)×100

The DS of fruits during storage was evaluated by observing the decay area on the fruit surface. Each fruit was scored from 1 to 5 based on the following disease rating scores: 1 = no fruit surface infection; 2 = slight (1–25% of the fruit surface infected); 3 = moderate (26–50% of the fruit surface infected); 4 = severe (51–75% of the fruit surface infected); and 5 = very severe (76–100% of the fruit surface infected). Equation 9 was used to calculate the DS index value.

9 $$DS\;\left(\%\right)=\frac{\sum \left(C\times i\right)}{M\times N}\times 100$$

Where DS is the percentage of disease severity, *C* is the number of fruits in the disease category, *i* is the numerical value of the disease category, *M* is the maximum value on the disease rating scale, and *N* is the number of fruits in all categories.^61^

### 2.5.10 Sensory Evaluation

A seven-member trained panel performed sensory analysis on strawberries at the beginning and every 5 days during room and cold storage. Sensory characteristics (color, firmness, sweetness, acidity, astringency, shriveling, off-flavor, taste, and overall quality) were regularly assessed by the same members, and only fruits without fungal decay were included in the evaluation. A 9-point hedonic scale (1 = dislike extremely; 5 = neither like nor dislike; 9 = like extremely) was used for sensory attributes, while a 5-point hedonic scale (1 = definitely would not buy; 3 = marketable; 5 = definitely would buy) was used for purchase intention.^8^

### 2.6 Statistical Analysis

Statistical analyses were conducted using SPSS (Version 23.0, IBM Corp., Armonk, NY, USA) and data were expressed as mean ± SD. To compare the physicochemical properties (moisture content, antioxidant activity, and color characteristics) of the two dextran samples, an independent-samples *t* test was performed. Prior to the *t* test, the normality of the data distribution was assessed using the Shapiro-Wilk test, and the homogeneity of variances was assessed via Levene’s test. For the inhibition zone data, one-way analysis of variance (ANOVA) was performed separately for each fungal strain to evaluate differences among EPS samples and the control. For the postharvest storage study, one-way and two-way ANOVAs were applied depending on the specific comparisons. To compare storage days within each treatment, one-way ANOVA was performed separately at 4 and 25 °C. For comparisons between treatments at the same storage day, two-way ANOVA was performed for Days 0 and 5, with treatment and storage temperature as the main factors and treatment × storage temperature as the interaction effect; all trials at both storage temperatures were included in the same analysis for each storage day. Since samples stored at 25 °C were not evaluated on Day 10, the Day 10 data at 4 °C were analyzed separately using one-way ANOVA to compare treatments. Tukey’s posthoc test was used for mean comparisons at a significance level of *p* < 0.05.

## 3 Results and Discussion

### 3.1 WKG Biomass Growth and Dextran Extraction Yield

After 30 consecutive cultivations, the average BIR (%) in the medium, including Figures, raisins, and brown sugar, was determined to be 8.61 ± 1.70 (%) and 7.72 ± 2.53 (%) (*n* = 3 biological replicates) for industrial and household WKG, respectively (*p* = 0.098). Low grain biomass growth is a prevalent problem during the fermentation of water kefir, despite the fact that getting grain biomass is crucial for the continuation and proliferation of grains and is also of considerable interest for the industrial application of the grains’ polysaccharide (dextran) as a novel biomaterial.^62^ Numerous studies on the growth in WKG biomass have been published in the literature, and they indicate that the increase can be either extremely low or very high, steady, or even negative. The results reported by Laureys and De Vuyst^63^ provide a clear example of this variability. They performed sequential fermentations (at 21 °C for 72 h) using three WKG from different households in a sterile medium containing unrefined cane sugar, Figure extract, and distilled water to examine the grain growth. The results demonstrated that one grain either did not increase its weight after each backslopping or did so very slowly; a second grain increased its weight until the fifth backslopping process and then decreased it after all subsequent backsloppings; and a third grain’s growth capability gradually decreased from the first backslopping to the last one. On the other hand, Lucena et al.^44^ reported a high grain growth ratio ranging from 85.02 to 125.88% in a medium containing 500 mL of mineral water and 35 g of brown sugar as substrates.

Many factors, including the frequency of inoculations, the size of WKG, the microbiota of grains, the available nutrients in the medium (e.g., the type of sugar or calcium content), fermentation duration, and temperature, have a significant impact on the biomass increase of WKG.^64^ These factors affect microbial growth and the synthesis of glucan, the primary EPS type in WKG, via altering glucansucrase activity. WKG is constructed of dextran, a glucose-based homopolysaccharide (glucan) with α-1,6-glycosidic connections in the main chain and α-1,3-glycosidic links in the ramifications.^65^ Previous studies have demonstrated that *Lentilactobacillus hilgardii* is the principal organism producing the dextran present in kefir grains. However, recent studies have indicated that *Liquorilactobacillus* (*L*.) *hordei*, *L. nagelii*, *Leuconostoc mesenteroides*, and *L. satsumensis* contribute to grain growth by producing dextran from sucrose.^54,64^ In this study, the dextran yield (%) was calculated using the weight of freeze-dried dextran/fresh WKG and was determined to be 3.83 ± 0.07 (%) and 5.20 ± 0.12 (%) (*n* = 3 biological replicates) for industrial and household WKG, respectively (*p* < 0.001). As far as is known, the only comparable study on dextran extraction from grains was conducted by Lucena et al.,^44^ who reported a yield of 1.80%. Previous studies obtained EPS in culture media or water kefir beverages fermented by specific dextran-producing LAB strains, including *Leuconostoc mesenteroides* and *L. hilgardii*,^66^ *L. hordei* and *L. mali*,^54^ and *Weissella confusa*.^67^

#### 3.2 Monosaccharide Composition of EPS

Chromatograms obtained from HPLC analysis of EPS demonstrate that the investigated EPS was a homopolysaccharide with a single peak. The retention times of various standard monosaccharides showed that only glucose was found in both EPS from industrial and household grains. Previous research presented similar results, with only a glucose peak identified in dextran^68,69^ and WKG^70^ samples. Using methylation analysis for structural characterization, Fels et al.^71^ demonstrated that WKGs contain a variety of glucose derivatives (a total of 81.10 g glucose/100 g WKG), with the majority being 1,6-linked glucose units and minor amounts of 1,3- and 1,4-linked glucose units.

### 3.3 SEM Observations and FTIR Results

Figure 1A,B depicts the microbial and micro structure of a cross-section belonging to grains. Industrial and household grains have similar general structural forms, with some variances. For instance, the porosity level of household grain is higher than that of industrial grain (Figure 1A1,B1). This means that industrial grain showed more compact morphological features in comparison to the household grain. In terms of microbial consortia, both grain types included a significant amount of yeast and a smaller number of bacilli (Figure 1A2,B2). SEM microphotographs suggested a denser spatial arrangement of yeast cells on the surface of industrial-type grains compared to household-type grains. *Lactococcus* organisms were not seen in the inner structure of both types of grains.

**Figure 1:**
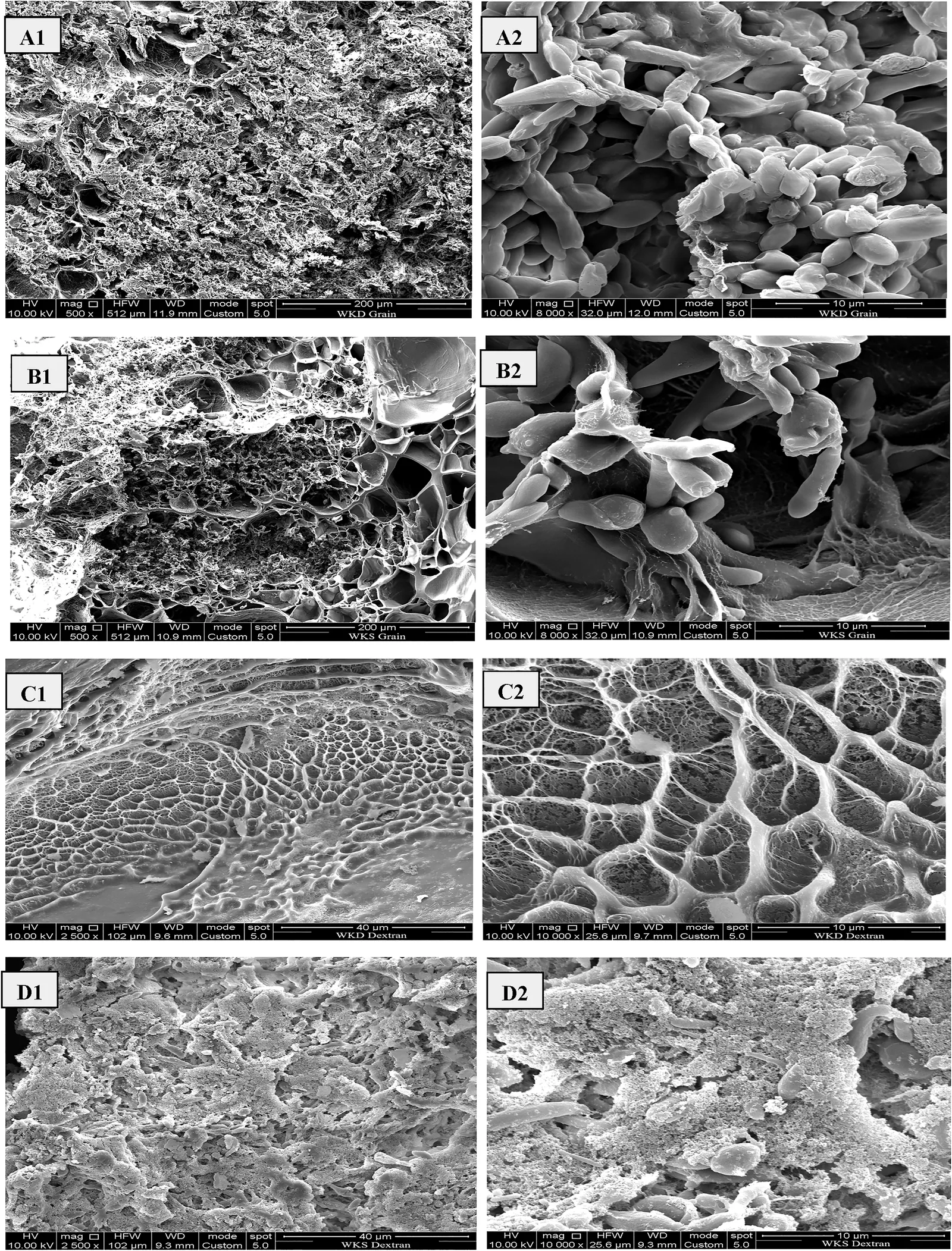
SEM images of industrial- and household-type water kefir grains and the dextran that was extracted from them. (A1) 500× magnified view of WKD, (A2) 8000× magnified view of WKD, (B1) 500× magnified view of WKS, (B2) 8000 × magnified view of WKS, (C1) 2500 × magnified view of dextran from WKD, (C2) 10,000 × magnified view of dextran from WKD, (D1) 2500 × magnified view of dextran from WKS, (D2) 10,000 × magnified view of dextran from WKS. Abbreviations: WKD: Industrial type grain, WKS: Household type grain.

Microstructural characteristics of both dextrans were shown in Figure 1C,D. Dextran from industrial WKG exhibited highly fibrous and compact morphological features (Figure 1C1,C2), whereas Figure 1D1,D2 depicted an uneven surface with abundant EPS layers for household WKG. The compact structure of the EPS illustrates dextran’s potential for biofilm formation or usage as a supportive agent or stabilizer.

FTIR spectroscopy revealed the polysaccharide structure of the extracellular compound produced by the LAB embedded in the WKG. Band assignments of the EPS materials were done according to ATR-FTIR spectral windows based on four different wavenumbers: 3600–3000 cm^–1^ (W1), 3000–2800 cm^–1^ (W2), 1700–1580 cm^–1^ (W3), and 1200–900 cm^–1^ (W4). W1 (Figure 2), an extremely broad window that contains the fundamental stretching modes of hydroxyl groups (O–H) due to water and carbohydrates; W2 bands attributed to the symmetric and antisymmetric stretching modes of C–H in methyl (CH_3_) and methylene (CH_2_) functional groups; W3, mainly associated with the O–H bending mode of absorbed water, with potential overlap from C=O stretching vibrations of carbonyl-containing compounds; and W4, a broad and intense band that holds the peaks mainly assigned to stretching modes of carbohydrate rings and side groups (−C–O–C, C–OH, C–H).^72^ The presence of two broad and intense bands in the W1 and W4 spectral ranges, which are mostly related to carbohydrate structure, confirmed that the tested compounds were polysaccharides.

**Figure 2:**
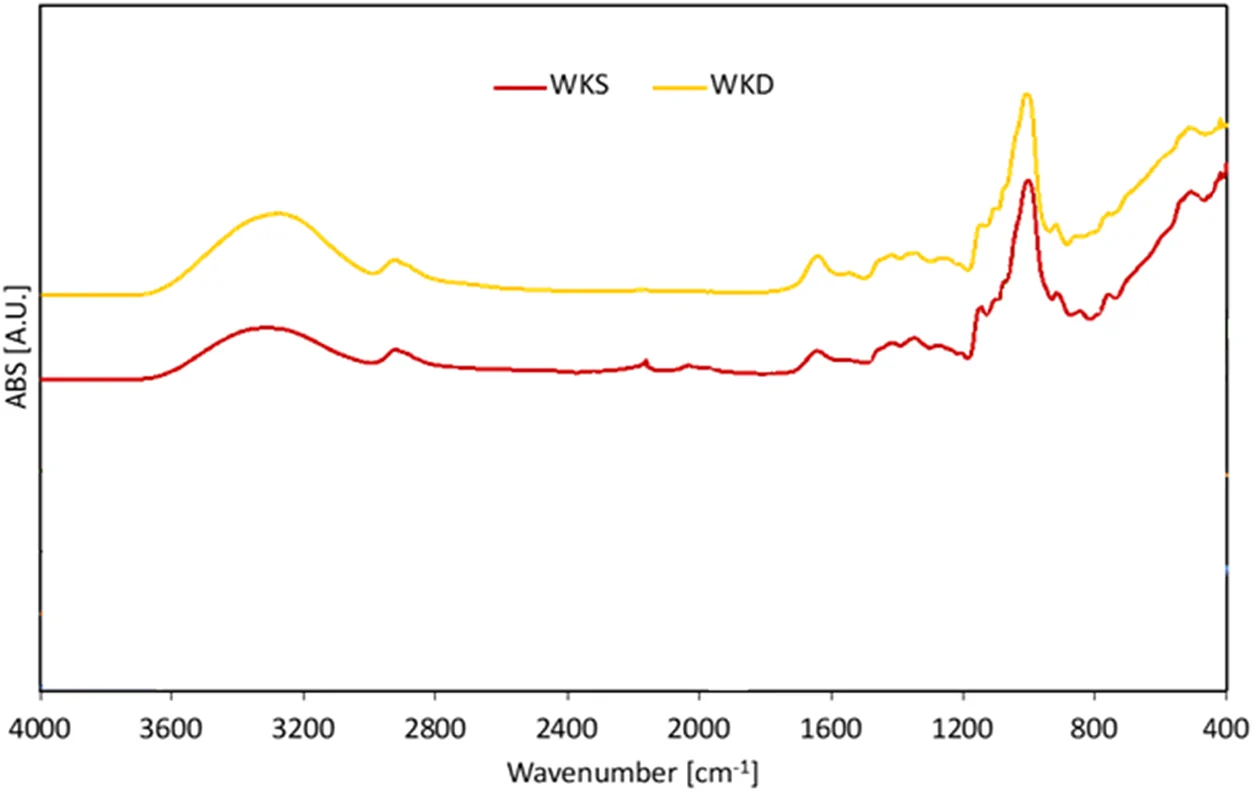
FTIR-ATR spectra of dextran exopolysaccharides. WKS: Dextran derived from household type WKG, WKD: Dextran derived from industrial type WKG.

The characteristic peak wavenumbers and their corresponding functional group assignments for WKS and WKD samples are detailed in Table 2. The primary, most intense band observed at 1015 cm^–1^ for WKS and 1012 cm^–1^ for WKD is attributed to C–O stretching vibrations characteristic of the polysaccharide structure. Notably, the distinct absorption band at 920 cm^–1^ confirmed the presence of C–O–C vibrations associated with α-(1→6) glycosidic linkages, typical for dextran. The highly similar spectral profiles observed for both samples are consistent with their polysaccharide nature and indicate a high degree of structural similarity between WKS and WKD.

**Table 2:** FTIR Peak Wavenumbers and Corresponding Band Assignments for WKS and WKD Samples

wavenumbers (cm^–1^)		
WKS	WKD	band assignments
3300	3300	O–H stretching vibrations of hydroxyl groups in water and carbohydrates
2925	2925	C–H symmetric and asymmetric stretching vibrations
1640	1640	O–H bending vibration of absorbed water and potential C = O stretching of carbonyl groups
1450	1450	C–H bending/deformation vibrations
1155	1155	antisymmetric C–O–C stretching vibrations associated with glycosidic linkages
1080	1080	vibrations involving C6–O6 stretching
1015	1012	C–O stretching vibrations associated with polysaccharide structure
920	920	C–O–C vibrations associated with α-(1→6) glycosidic linkages

Davidović et al.^66^ used FTIR to investigate the main structural groups of pure dextrans produced by three LAB isolates (*Leuconostoc mesenteroides* (T1 and T3) and *Lactobacillus hilgardii*) isolated from WKG. Two broad and intense signals were detected in three dextran materials, similar to those identified within the present study. The first is intense and broad at 3200–3500 cm^–1^, originating from the O–H stretching vibrations due to the impact of hydrogen bonds, which is characteristic for polysaccharide molecules. The latter broad signal, which originates from C–O–C and C–O at 1200–1000 cm^–1^, confirms that the substances under examination are polysaccharides and shows the presence of the distinctive bonds of carbohydrates.

### 3.4 Moisture, Antioxidant Activity and Color Properties

The moisture content of nonfreeze-dried EPSs from WKD and WKS was 80% (dry matter 20%) and 85.26% (dry matter 14.74%), respectively (Table 3, *p* = 0.04). The significant differences in dextran’s dry matter content may have an impact on grain structure due to variations in protein and mineral content. The dry matter results indicate that dextran from WKD displays a denser and more compact structure than dextran from WKS, as corroborated by the SEM images presented in section 3.2 The moisture content of dextran EPS and fresh grains are nearly similar. Gökırmaklı and Güzel-Seydim^49^ reported a moisture level of 82.5% in WK grain, which is remarkably similar to what was found in this study.

**Table 3:** Moisture, Antioxidant Activity and Colour Characteristics of Dextrans Derived from Industrial and Household WKG^a^

characteristics	dextran from WKD	dextran from WKS
moisture (%)	80.00 ± 0.28^b^	85.26 ± 0.36^a^
Antioxidant Activity Values		
DPPH (%)	49.30 ± 0.42^b^	57.40 ± 0.56^a^
ABTS (%)	51.80 ± 1.13^b^	60.70 ± 0.98^a^
FRAP (μmol TE/L)	56.60 ± 0.84^b^	63.70 ± 0.98^a^
Color Parameters		
*L*	74.28 ± 2.83^a^	75.09 ± 2.63^a^
*a*	2.62 ± 0.57^a^	2.84 ± 0.23^a^
*b*	14.54 ± 1.09^a^	14.57 ± 1.05^a^
C*_ab_	14.77 ± 1.15^a^	14.85 ± 1.06^a^
*H*°	79.80 ± 1.68^a^	78.93 ± 0.79^a^
Δ*E*	29.63 ± 1.71^a^	28.97 ± 2.04^a^
WI	70.27 ± 2.02^a^	70.96 ± 2.20^a^

a The mean values displayed with different letters in the same row differ statistically (*p* < 0.05). DPPH and ABTS values were calculated as the % inhibition capacity of dextran extracts in scavenging DPPH and ABTS radicals, respectively. Abbreviations: WKD: Industrial type grain, WKS: Household type grain, TE: Trolox equivalent, C*_ab_: Chroma, *H°*: Hue, Δ*E*: Total color difference, WI: Whiteness index

Three different methods were employed in this study to evaluate the antioxidant capacity of dextrans because AA can arise through various mechanisms and reactions, including radical scavenging, reductive capacity, chain initiation prevention, and binding of transition metal catalysts.^51^ Therefore, the ability of dextran to scavenge DPPH and ABTS free radicals, as well as the ferric reducing antioxidant power (FRAP) assay, were tested. It is worth noting that in all three methods assessed, dextran extracted from household-type grain displayed greater antioxidant activity than dextran extracted from industrial-type WKG (Table 3, *p* = 0.004 for DPPH, *p* = 0.014 for ABTS, and *p* = 0.016 for FRAP). However, as Table 3 illustrates, both types of dextran exhibited strong scavenging activity against DPPH and ABTS radicals, as well as significant reductive capability of a ferric-TPTZ complex from Fe^3+^ to Fe^2+^. The FRAP method, which used the lowest dose of dextran (0.3 mg), yielded the highest AA of the three methods tested. Both dextrans exhibited reduction capacity in a WKG-type-dependent manner, with antioxidant values of 63.70 μmol TE/L for EPS from the household type and 56.60 μmol TE/L for the EPS obtained from the industrial-type grain. At a concentration of 0.5 mg EPS, dextran from industrial-type grain had a scavenging ability on the ABTS free radical of 51.80%, which was lower than that of household-type grain (60.70%). Despite using a higher dosage of dextran (3 mg) in the test, DPPH inhibition (from 49.30% to 57.40%) was shown to be lower than values obtained for ABTS inhibition.

These findings are generally consistent with previous reports demonstrating that microbial dextrans can exhibit considerable antioxidant activity, although the magnitude of this activity varies depending on the dextran source, molecular characteristics, assay conditions, and concentration. For example, dextran produced by *Lactobacillus kunkeei* AK1 showed 46.23 ± 1.52% DPPH radical-scavenging activity at 4 mg/mL and 91.6 ± 1.98% ABTS radical-scavenging activity at 8 mg/mL.^73^ The latter value was also reported to be comparable to the antioxidant activity of ascorbic acid under the tested conditions.^73^ Similarly, an in vitro-synthesized dextran from *Leuconostoc pseudomesenteroides* exhibited 29.56 ± 1.82% DPPH, and 40.46 ± 1.61% ABTS scavenging activity at 6 mg/mL.^74^ In another comparative study, dextran produced by *Leuconostoc pseudomesenteroides* strain DRP-5 showed 66.52 ± 2.76% DPPH and 87.82 ± 3.65% ABTS scavenging activity at 7.0 mg/mL.^75^ Overall, these findings indicate that the antioxidant activities observed for the household- and industrial-type WKG dextrans are comparable to those reported for other microbial dextrans, although direct comparisons should be interpreted cautiously due to differences in dextran concentration and experimental conditions. Although literature reports indicate that understanding of the precise antioxidant mechanisms of microbial EPS remains limited, the observed variations in antioxidant activity among various dextrans may be attributed to their monosaccharide composition, type of glycosidic linkages, molecular weight, hydrogen-donating ability, branching structure, and the presence of functional groups such as polyphenolic, hydroxyl, carboxyl, and sulfonyl moieties.^50^

The colors of foods, and particularly biopolymers used in food coatings because of their film-forming properties, have an important impact on customer acceptability. Table 3 indicates that the *L*, *a*, *b*, C*_ab_, *H*°, WI, and Δ*E* values in dextrans from household- and industrial-type kefirs did not differ significantly. The results showed that dextran from household-type WKG had the highest values of *L*, *a*, *b*, C*_ab_, and WI, whereas dextran from industrial-type WKG had greater values of *H*° and WI. The color similarity of dextran samples is illustrated by highly similar Δ*E* values (28.97 and 29.63), which describe the level of color difference between two samples. The low + *a* values suggest that dextrans have minimal redness that is not perceptible to the human eye. The *L* and WI values, while significantly below 100 yet beyond 70, suggest that the samples are not entirely white but primarily whitish. The b values range from 14.54 to 14.57, indicating that EPSs possess a slight yellow color. These findings suggest that dextrans have a white look with yellow spots. C*_ab_ indicates color vividness or saturation in a range from gray (C*_ab_ = 0) to pure color (C*_ab_ = 100). The *H*° parameter expresses the color tones by indicating the degree of the dominating spectral components. For the interpretation, the results are expressed in angles that vary from 0 to 360°, beginning with 0 or 360° (red), 90° (yellow), 180° (green), and 270° (blue).^76^ Dextrans had low average C*_ab_ values (Table 3), indicating that the purity or intensity of their color is low. Furthermore, the H° values of both dextrans were in the yellow spectral zone. According to all color parameters obtained, the dextrans obtained in the present study appear to be a faint (light) yellow. WKGs are translucent and typically white to gray in color.^49^ However, depending on the type of sucrose used and the fruit and vegetables added to the growth medium, the grains’ color might range from white to yellowish.^64^ In this regard, it can be said that raisins, Figures, and brown sugar, the substrates used in this study to increase biomass in WKGs, result in the grain color shifting from white to light yellow. The literature contains investigations demonstrating that dextran can be obtained in various colors. Dextran produced by *Leuconostoc* spp., for example, has been found to be white,^77^ although solutions containing different concentrations of dextran (50 to 10,000 mg/L) range in color from pale yellow to dark brown.^78^

### 3.5 Antifungal Activity of EPS

This study utilized eight molds, including common postharvest pathogens, to assess the antifungal efficacy of dextran samples using broth microdilution and disk diffusion methods, with results summarized in Table 4. As shown in Table 4, dextran from household type WKG had greater antifungal activity than dextran from industrial type WKG, with larger zones of inhibition (mm) and lower MIC values against all tested fungal pathogens (*p* ***<*** 0.05). The zone of inhibition results showed that dextran from household-type grain was equally effective as antibiotic disks (positive control) against *Penicillium italicum* ML332 (*p* = 0.94) and *Penicillium echinulatum* ML291 (*p* = 0.33) and was statistically in the same category (*p >* 0.05). Except for *Alternaria alternata* ML356, both EPS exhibited potent antifungal activity against seven species of fungi, with MIC values ranging from 0.1 to 10 mg/mL. The MIC value, or the lowest concentration at which no change in absorbance indicates no fungal growth, was 100 mg/mL for *Alternaria alternata* ML356 in dextran from household-type grain, whereas it was above 100 mg/mL for EPS obtained from industrial-type WKG. The MIC and inhibition zone test results are in agreement. According to both tests, *Penicillium* sp. was the most sensitive mold genus to dextran EPS, followed by *Aspergillus* sp. and *Alternaria alternata* ML356.

**Table 4:** Antifungal Activity of the Dextran Exopolysaccharides^a^

	diameter of inhibition zones (mm)							
	mold strains							
	*Aspergillus* *fumigatus*	*Aspergillus* *flavus*	*Aspergillus* *brasiliensis*	*Penicillium* *italicum*	*Penicillium* *echinulatum*	*Penicillium* *digitatum*	*Penicillium* *expansum*	*Alternaria* *alternata*
EPS samples	ATCC-204305	BCCM/MUCL18903	BCCM/MUCL29039	ML332	ML291	DSM2750	DSM62841	ML356
D-WKS	14.77 ± 0.44^b^	11.77 ± 0.89^b^	12.67 ± 1.81^b^	23.01 ± 1.42^a^	22.01 ± 1.24^a^	16.13 ± 1.14^b^	18.35 ± 1.59^b^	4.90 ± 0.28^b^
D-WKD	11.00 ± 0.81^c^	9.75 ± 0.86^c^	9.56 ± 1.22^b^	13.54 ± 1.10^b^	11.77 ± 0.74^b^	10.59 ± 0.68^c^	11.34 ± 0.38^c^	1.43 ± 0.12^c^
cycloheximide	24.06 ± 1.13^a^	19.98 ± 0.62^a^	28.22 ± 1.63^a^	22.38 ± 1.79^a^	20.69 ± 1.08^a^	20.60 ± 1.19^a^	23.36 ± 1.67^a^	15.01 ± 1.19^a^
	minimum inhibitory concentration (MIC, mg/mL)							
D-WKS	1	1	1	0.1	0.1	0.1	0.1	100
D-WKD	1	10	10	1	1	1	1	>100
cycloheximide	0.1	0.1	0.1	0.01	0.01	0.01	0.01	1

a The mean values displayed with different letters in the same column differ statistically (*p* < 0.05). Abbreviations: D-WKS: Dextran obtained from household type grain, D-WKD: Dextran obtained from industrial type grain.

Unlike kefiran (the EPS of milk kefir grains) whose antimicrobial activity is well documented,^79^ little is known about antimicrobial activity of EPS from WKG. Those studies that have been done have mostly concentrated on dextran’s antibacterial properties against Gram-positive and Gram-negative bacteria; its antifungal characteristics have received less attention. According to these studies, dextran derived from WKG is identified as a valuable antimicrobial EPS. Lucena et al.^44^ concluded that Gram-negative bacteria (*Escherichia coli*, MIC: 2.5 mg/mL) were more sensitive to the presence of EPS from WKG than Gram-positive bacteria (*Staphylococcus aureus*, MIC: 5 mg/mL). Similar findings were reported against *Escherichia coli* (MIC: 2.0 mg/mL) and *Staphylococcus aureus* (MIC: 3.0 mg/mL) by Ye et al.,^80^ who assessed the antibacterial activity of dextran from *Leuconostoc pseudomesenteroides* YB-2. Furthermore, by increasing the dextran concentration from 5 to 30 mg/mL, the zone of inhibition increased from 4.79 to 9.10 mm against *Staphylococcus aureus* and from 9.11 to 11.94 mm against *Escherichia coli*.

Although some studies have examined the antimicrobial properties of EPSs derived from microbial cells, the mechanism of action remains unclear. Several possible mechanisms have been proposed for the antimicrobial activity of the EPS from microorganisms, including impairing cell division, disrupting the cell wall and cytoplasmic membrane, and decomposing DNA.^50,80^ EPSs can interact efficiently with both Gram-negative and Gram-positive bacteria depending on their cell membrane permeability, affecting the respiratory chain and cell division machinery and finally causing cell death.^81^ Han et al.^82^ further highlighted the antimicrobial capacity of EPS in changing the permeability of bacterial cell walls and membranes and proposed that exopolysaccharides might act as a barrier that prevents the import of nutrients and inhibits bacterial growth. Mohamed et al.^83^ reported another mechanism of purified EPSs antimicrobial activity, stating that anionic polysaccharides, such as sulfated polysaccharides, exhibit antimicrobial activity through a variety of mechanisms, including chelation activities and the deprivation of metals, trace elements, or essential nutrients, that limit microbial growth. Finally, more research is needed to understand the mechanism of dextran EPS’s antimicrobial effect on pathogenic bacteria and fungi.

### 3.6 Strawberry Storage Studies

#### 3.6.1 Weight Loss (WL)

The weight is a critical quality indicator of fruits and vegetables. Agricultural producers and commercial enterprises distributing fruits and vegetables to the market aim to minimize WL during storage. The primary reason for the desire is that as the WL of the product increases during storage, the seller’s potential profit from its sale correspondingly diminishes. The weight losses in fruit are primarily attributed to an elevated rate of transpiration and respiration processes.^2^ Strawberry fruit is prone to quick water loss due to its thin skin, which causes shriveling, dryness, and a darkening of the red color. Edible coatings function as barriers that limit water transfer and protect the fruit skin from mechanical damage. They also seal minor wounds, which helps to postpone dehydration.^60^ Figure 3A presents the weight loss (measured in grams) for different treatments during a shelf life study conducted over 10 days under cold storage conditions and 5 days under ambient conditions.

**Figure 3:**
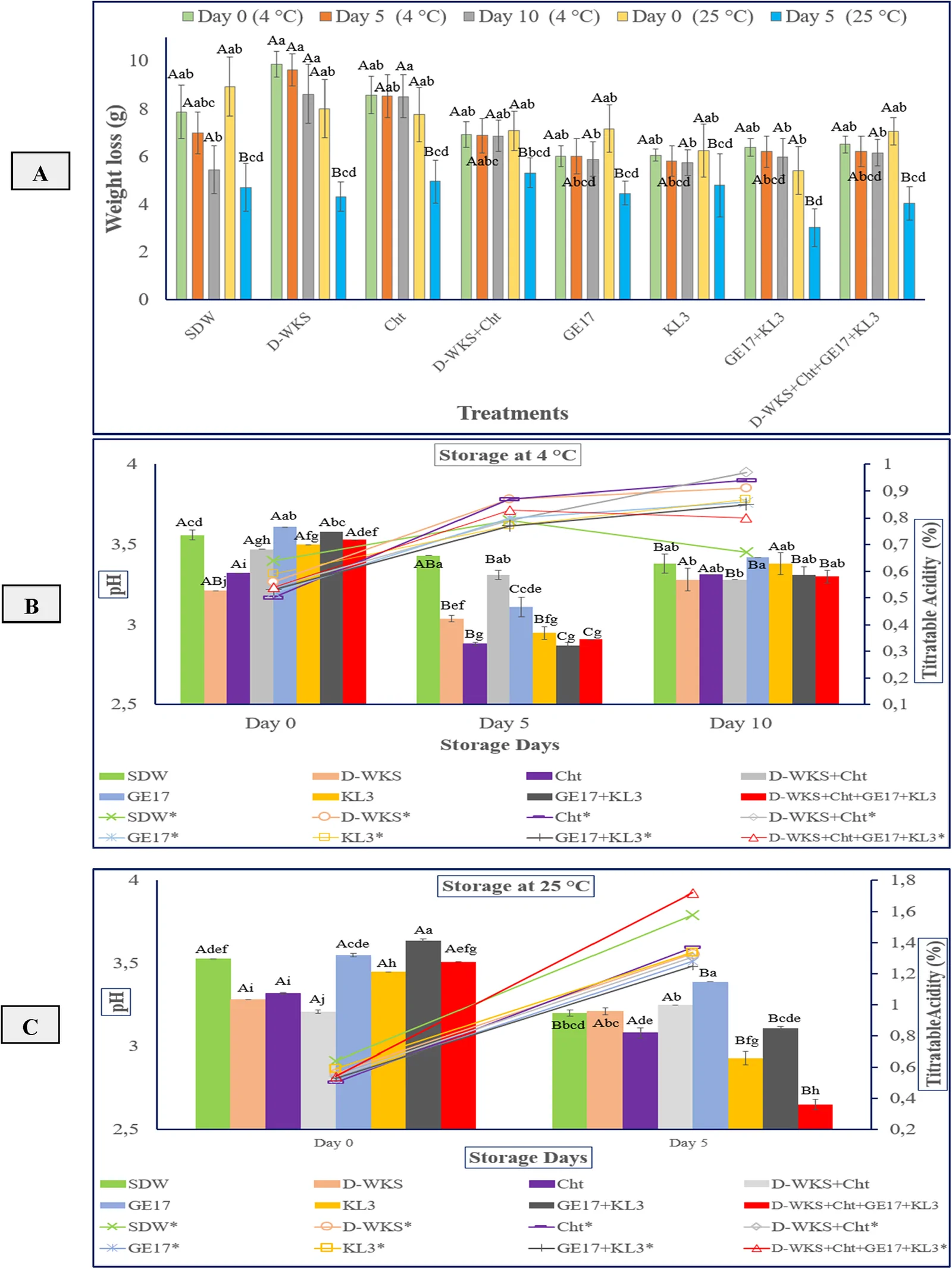
Variations in strawberry fruit weight (A), pH (B at 4 °C and C at 25 °C), and titratable acidity (B at 4 °C and C at 25 °C) during ambient and cold storage. Abbreviations: Symbols labeled with an asterisk (*) represent the TA value. SDW: Sterile distilled water, D-WKS: Dextran derived from household type grain, Cht: Chitosan, GE17: *Aureobasidium pullulans* GE17, KL3: *Meyerozyma guilliermondii* KL3, uppercase superscripts indicate significant differences among various storage days within the same treatment (with groups stored at 4 and 25 °C evaluated independently for each treatment), lowercase superscripts indicate significant differences among all treatments on the same storage day (where all trials comprising samples stored at 4 and 25 °C were analyzed simultaneously). In Figure 3B,C, letters above the bars represent statistical differences for pH values.

The weight of fruits was consistently decreased over time across all treatments; however, no statistical differences were seen in WL values in the storage duration of the treatments at 4 °C (*p* > 0.05). Conversely, weight losses were observed to be significant within each experiment during the storage period at 25 °C (*p* < 0.05). Figure 3A clarifies that the WL values (in grams) on the fifth day of storage at 25 °C were significantly higher compared to those observed on the fifth day at 4 °C. At the end of the both storage conditions, the highest WL level was observed for the control sample as 30.83 and 47.25% for storage at 4 and 25 °C, respectively. The lowest percentage was determined for treatment Cht as 0.58%, followed by D-WKS + Cht at 0.72%, GE17 at 2.32%, KL3 at 5.11%, D-WKS + Cht + GE17 + KL3 at 5.37%, GE17 + KL3 at 6.42%, and D-WKS at 12.47% after 10 days of storage for strawberry fruits maintained at 4 °C. These results are comparable with those of Badawy et al.,^84^ who reported a 14.30% weight loss in uncoated control strawberries after 7 days at 4 °C, whereas coating formulations of chitosan alone or combined with gelatin, starch, and essential oils (thymol/geraniol) limited this loss to 0.61–2.81%. The authors suggested that the higher viscosity of chitosan-starch solutions likely produced a thicker coating, thereby further reducing moisture loss.

For storage at 25 °C, fruits treated with KL3 had the lowest WL (23.20%), followed by D-WKS + Cht (24.85%), Cht (36.08%), GE17 (37.88%), D-WKS + Cht + GE17 + KL3 (42.85%), GE17 + KL3 (44.28%), and D-WKS (46.06%). Upon evaluating the data from both storage conditions collectively, it is evident that chitosan significantly mitigates weight loss in fruits, and the impact of D-WKS, which is minimal when utilized individually as a coating material, becomes noticeable when paired with chitosan. KL3 yeast is considered the coating biomaterial that most significantly reduces the WL ratio under ambient storage conditions. This reduction is believed to result from a combination of mechanisms of yeast, including lytic enzyme activity, production of volatile organic compounds, inhibition of fungal pathogen spore germination, and competition for nutrition and space.^11^ This study’s findings align with Bal,^60^ who noted that chitosan coatings on strawberries can diminish water loss during storage by serving as a semipermeable barrier to oxygen, carbon dioxide, and moisture, thus lowering respiration and water loss.

#### 3.6.2 pH and Titratable Acidity

The pH level and TA are critical quality parameters in strawberries, as they directly influence the fruit’s flavor profile, microbial stability, and shelf life. Furthermore, in strawberries, the balance between pH and TA plays a vital role in determining the characteristic sweet-tart flavor that influences consumer preference. As indicated in Figure 3B, in all treatments stored at 4 °C, a reduction in pH values was noted at the end of the storage period when compared to the starting level, while an increase in TA levels was recorded (*p* < 0.05). A similar trend was observed in strawberries maintained at 25 °C (Figure 3C); however, the reduction in pH and the elevation in TA were markedly greater than those observed at 4 °C. Namely, after storage at 4 °C, the pH and TA values were measured to be between 3.28 and 3.42 and 0.67–0.97% (Supporting 1), respectively. In contrast, following storage at 25 °C, the pH and TA values ranged from 2.65 to 3.39 and 1.25 to 1.72%, respectively. In summary, 25 °C storage (ambient condition) led to more drastic changes in both pH and TA. The most significant drop in pH and the highest increase in acidity were observed here, indicating rapid metabolic activity or spoilage. Four °C storage (cold storage condition) effectively slowed down the changes in pH and TA, maintaining the chemical stability of the fruit for up to 10 days. In terms of acidity preservation at 4 °C, the Cht application stood out as the most stable treatment, maintaining a nearly constant pH level that shifted only from 3.32 to 3.31 over the 10-day period. On the other hand, the sharpest decline at 25 °C was observed in the D-WKS+Cht+GE17+KL3 combination, which saw a significant pH drop of 0.86 units (from 3.51 on day 0 to 2.65 by day 5), suggesting that this complex coating might not be ideal for room-temperature storage. In alignment with the sharpest pH decline, the D-WKS+Cht+GE17+KL3 treatment also exhibited the highest increase in TA at 25 °C, with a substantial rise of 1.18 units, moving from 0.54% on day 0 to 1.72% by day 5. The pronounced acidification observed in the D-WKS+Cht+GE17+KL3 treatment under ambient storage may be associated with the combined effects of its multiple coating components under the more demanding conditions at 25 °C. Unlike the individual or simpler coating formulations, this treatment contained two biocontrol agents together with D-WKS and chitosan, which may have resulted in interactions among the coating components and altered the physicochemical and biological conditions at the fruit surface. Such interactions may have affected gas exchange, microbial activity, or the metabolism of organic acids during ambient storage. Therefore, the marked acidification observed in this multicomponent formulation may reflect a formulation-specific response arising from the interaction of multiple coating components under ambient conditions. However, the precise mechanism underlying this response cannot be conclusively determined from the present data and requires further investigation.

Consistently, increases in titratable acidity (TA) during strawberry storage have been documented by Ali et al.,^85^ Caleb et al.,^86^ and Aamer et al.^87^ Although the prevailing literature generally reports an increase in pH during storage due to the metabolic consumption of organic acids during respiration,^88,89^ a simultaneous decrease in pH and increase in TA has also been reported in strawberries under certain postharvest conditions. Tumbarski et al.^90^ observed an increase in TA accompanied by a decrease in pH in strawberries treated with edible coatings during 16 days of storage at 4 °C. Their analysis of organic acids further demonstrated that the concentration of citric acid, one of the predominant organic acids in strawberries, continuously increased throughout the storage period, consistent with the observed increase in TA and decrease in pH. Similarly, Atci et al.^91^ reported a decrease in pH together with an increase in TA in strawberries during refrigerated storage, supporting the occurrence of this inverse relationship during postharvest storage. In the present study, the simultaneous increase in TA and decrease in pH may be partly explained by a concentration effect associated with postharvest water loss. As water is lost from the fruit through transpiration and evaporation, the concentration of nonvolatile organic acids per unit fresh mass may increase, thereby contributing to higher TA and lower pH values. This explanation is further supported by the greater moisture loss observed at 25 °C compared with 4 °C, as shown in Figure 3A (*p* < 0.05). Notably, even at the end of the shorter ambient storage period (Day 5), all treatments exhibited higher TA values and lower pH values than those observed at the end of the longer cold storage period (Day 10). The corresponding numerical values and statistical analyses for TA are provided in Supporting File 1, while the pH values and their statistical comparisons are presented in Figure 3B,C. These findings suggest that the greater water loss and accelerated postharvest physiological changes under ambient conditions may have contributed to the concentration of organic acids, resulting in higher TA and lower pH values.

#### 3.6.3 °Brix, Browning Index, Firmness and Total Phenolic Content

The °Brix level serves as an indicator of the total soluble solids within the fruit matrix, corresponding to the percentage of sucrose content at a standard temperature of 20 °C.^92^ °Brix serve as a key indicator of fruit maturity, sweetness, and postharvest quality, generally increasing as fruit ripens due to starch conversion. It predominantly reflects sugar content (fructose, glucose, and sucrose) alongside organic acids, amino acids, and minerals. A high, stable °Brix value indicates better taste, better maturity, and higher consumer acceptance.^93^

In terms of cold storage (4 °C), the control group (SDW) experienced a substantial TSS loss of 48.25% (*p* *<* 0.05) (Table 5). Conversely, the Cht treatment remarkably restricted this decline to only 7.21%, demonstrating the highest preservation efficacy. The combination of D-WKS+Cht also showed high retention with only a 14.43% reduction at 4 °C. In contrast, treatments involving only biocontrol yeasts (GE17, KL3) and their combination (GE17+KL3) showed significantly higher losses, ranging between 27.11 and 28.67% at the same temperature (*p* *<* 0.05). Regarding ambient conditions (25 °C), the SDW group lost 58.08% of its initial Brix content. Once again, the Cht coating proved to be the most effective treatment, limiting the relative loss to 24.79%. While the D-WKS+Cht group also mitigated the loss (37.32%) at 25 °C, the yeast-only treatments (GE17, KL3) and the GE17+KL3 combination suffered severe losses exceeding 48.63 to 54.33%, thereby closely mirroring the decline observed in the control (*p* *<* 0.05). Overall, these findings indicate that while low-temperature storage (4 °C) is fundamental for preserving strawberry quality, the application of Cht and D-WKS coatings provides a synergistic effect that remains superior even under ambient conditions (25 °C). Consequently, these coatings effectively delay the metabolic depletion of sugars, extending the shelf life and maintaining the sweetness profile of the fruit during both cold and ambient distribution chains.

**Table 5:** Changes in Some Quality Characteristics of Coated Strawberries under Cold (4 °C) and Ambient (25 °C) Storage Conditions^a^

	experiments	SDW	D-WKS	Cht	D-WKS+Cht	GE17	KL3	GE17 + KL3	D-WKS+Cht+GE17+KL3
**analysis**	**storage days**	**storage at 4 °C**							
Brix (%)	Day 0	6.30 ± 0.20^Aabc^	5.76 ± 0.05^Ac^	6.10 ± 0.34^Abc^	5.96 ± 0.05^Abc^	5.86 ± 0.11^Abc^	6.53 ± 0.05^Aabc^	5.93 ± 0.05^Abc^	6.06 ± 0.11^Aabc^
	Day 5	5.86 ± 0.11^Bab^	5.63 ± 0.05^Abc^	6.00 ± 0.10^Aa^	5.73 ± 0.05^Babc^	4.83 ± 0.15^Bd^	5.66 ± 0.05^Babc^	5.80 ± 0.10^Aabc^	5.46 ± 0.05^Bc^
	Day 10	3.26 ± 0.15^Ce^	4.70 ± 0.20^Bc^	5.66 ± 0.15^Aa^	5.10 ± 0.00^Cb^	4.23 ± 0.23^Cd^	4.76 ± 0.11^Cbc^	4.23 ± 0.05^Bd^	4.96 ± 0.05^Cbc^
BI (A_420_)	Day 0	0.497 ± 0.00^Ca^	0.435 ± 0.00^*Cc*^	0.494 ± 0.00^Ca^	0.441 ± 0.00^*Cc*^	0.389 ± 0.00^Cef^	0.412 ± 0.00^Cd^	0.457 ± 0.00^Cb^	0.466 ± 0.00^Cb^
	Day 5	0.540 ± 0.00^Bgh^	0.457 ± 0.00^Bk^	0.677 ± 0.00^Bc^	0.563 ± 0.00^Be^	0.541 ± 0.00^Bg^	0.712 ± 0.00^Ba^	0.556 ± 0.00^Bf^	0.492 ± 0.00^Bi^
	Day 10	0.759 ± 0.00^Ab^	0.524 ± 0.00^Ah^	0.685 ± 0.00^Ad^	0.622 ± 0.00^Af^	0.571 ± 0.00^Ag^	0.738 ± 0.00^Ac^	1.070 ± 0.00^Aa^	0.679 ± 0.00^Ae^
firmness (N)	Day 0	7.34 ± 0.06^Ag^	7.44 ± 0.05^Aefg^	7.63 ± 0.05^Abc^	7.68 ± 0.04^Aab^	7.62 ± 0.05^Abc^	7.60 ± 0.04^Abc^	7.44 ± 0.01^Aefg^	7.36 ± 0.01^Afg^
	Day 5	6.98 ± 0.04^Bf^	7.31 ± 0.04^Bcde^	7.55 ± 0.04^Aab^	7.58 ± 0.04^Aa^	7.42 ± 0.09^Bbc^	7.41 ± 0.04^Bbcd^	7.26 ± 0.05^Bde^	7.20 ± 0.04^Be^
	Day 10	6.20 ± 0.07^Cd^	6.96 ± 0.01^Cb^	7.36 ± 0.07^Ba^	7.31 ± 0.06^Ba^	6.87 ± 0.04^Cbc^	6.88 ± 0.04^Cbc^	6.80 ± 0.05^*Cc*^	6.80 ± 0.04^*Cc*^
TFC (mg GAE/100 g)	Day 0	152.73 ± 3.52^Aab^	152.19 ± 4.88^Aabc^	153.33 ± 4.74^Aab^	156.94 ± 6.53^Aab^	160.80 ± 2.60^Aab^	149.83 ± 3.38^Aabc^	153.95 ± 0.42^Aab^	165.31 ± 4.71^Aa^
	Day 5	147.43 ± 3.93^ABabc^	146.45 ± 5.35^Aabc^	140.20 ± 13.07^Aabcd^	151.65 ± 5.65^Aab^	154.44 ± 1.83^Aa^	143.77 ± 4.70^ABabcd^	147.25 ± 0.28^Babc^	159.13 ± 3.61^Aa^
	Day 10	137.44 ± 2.55^Bab^	140.21 ± 5.04^Aab^	133.98 ± 11.73^Ab^	145.11 ± 4.43^Aab^	145.73 ± 0.83^Bab^	136.24 ± 3.60^Bb^	139.11 ± 0.80^Cab^	150.94 ± 5.28^Aa^
		**storage at 25 °C**							
Brix (%)	Day 0	7.30 ± 1.38^Aa^	6.66 ± 0.05^Aabc^	5.93 ± 0.05^Abc^	6.86 ± 0.05^Aabc^	6.93 ± 0.15^Aab^	6.70 ± 0.10^Aabc^	6.36 ± 0.11^Aabc^	6.70 ± 0.00^Aabc^
	Day 5	3.06 ± 0.05^Bgh^	3.53 ± 0.15^Bf^	4.46 ± 0.15^Be^	4.30 ± 0.20^Be^	3.56 ± 0.15^Bf^	3.06 ± 0.11^Bgh^	2.93 ± 0.11^Bh^	3.33 ± 0.05^Bfg^
BI (A_420_)	Day 0	0.427 ± 0.00^Bc^	0.441 ± 0.01^Bc^	0.438 ± 0.00^Bc^	0.386 ± 0.00^Bef^	0.270 ± 0.00^Bh^	0.397 ± 0.00^Be^	0.347 ± 0.00^Bg^	0.376 ± 0.00^Bf^
	Day 5	0.698 ± 0.00^Ab^	0.587 ± 0.00^Ad^	0.696 ± 0.00^Ab^	0.536 ± 0.00^Ah^	0.413 ± 0.00^Al^	0.473 ± 0.00^Aj^	0.472 ± 0.00^Aj^	0.552 ± 0.00^Af^
firmness (N)	Day 0	7.49 ± 0.01^Ade^	7.34 ± 0.01^Ag^	7.47 ± 0.02^Adef^	7.56 ± 0.02^Acd^	7.65 ± 0.03^Aabc^	7.74 ± 0.03^Aa^	7.46 ± 0.00^Adef^	7.38 ± 0.00^Afg^
	Day 5	6.07 ± 0.04^Bi^	6.72 ± 0.05^Bh^	7.04 ± 0.08^Bf^	7.04 ± 0.02^Bf^	6.79 ± 0.06^Bgh^	6.94 ± 0.04^Bfg^	6.71 ± 0.01^Bh^	6.70 ± 0.01^Bh^
TFC (mg GAE/100 g)	Day 0	144.95 ± 3.81^Abc^	153.46 ± 4.56^Aab^	142.18 ± 4.93^Abc^	150.61 ± 3.71^Aabc^	133.61 ± 5.55^Ac^	142.45 ± 7.35^Abc^	156.49 ± 3.07^Aab^	154.36 ± 6.19^Aab^
	Day 5	129.43 ± 4.39^Bcd^	144.02 ± 4.72^Babcd^	131.67 ± 5.68^Bbcd^	142.24 ± 2.84^Babcd^	122.45 ± 3.92^Ad^	132.22 ± 9.29^Abcd^	141.40 ± 1.65^Babcd^	143.60 ± 3.71^Aabcd^

a Abbreviations: BI: Browning index, TFC: Total phenolic content, SDW: Sterile distilled water, D-WKS: Dextran derived from household type grain, Cht: Chitosan, GE17: *Aureobasidium pullulans* GE17, KL3: *Meyerozyma guilliermondii* KL3, uppercase superscripts indicate significant differences among various storage days of the same treatment, with groups at 4 and 25 °C evaluated independently for the identical treatment, significant differences between all of the treatments on the same day are indicated by lowercase superscripts (all trials included in the study, which comprised samples stored at 4 and 25 °C, were analyzed at once).

The browning index (BI), which quantifies pigment intensity through spectrophotometric absorbance, serves as a critical indicator of enzymatic browning resulting from the oxidation of phenolic compounds.^94^ In fresh fruits like strawberries, monitoring BI is essential for evaluating postharvest quality, shelf life, and consumer acceptability, particularly under different storage temperatures and coating treatments. BI was monitored as a vital indicator of the enzymatic browning and overall postharvest quality of strawberries during storage at 4 and 25 °C. Regardless of the treatment, BI values significantly increased over the storage period in all samples (*p* *<* 0.05), as indicated by the uppercase superscripts (Table 5). The progression of browning, primarily driven by polyphenol oxidase (PPO) activity and nonenzymatic processes including ascorbic acid degradation, polymerization, and Maillard reactions, was notably more rapid and intense at 25 °C compared to 4 °C storage.^95^ For instance, the control group (SDW) reached a BI of 0.698 (A_420_) by day 5 at 25 °C, whereas it only reached 0.540 (A_420_) at 4 °C during the same interval. The efficacy of the applied coatings was evaluated by observing the net increase in browning index (ΔBI) from the initial day to the end of the storage period, rather than comparing absolute absorbance values. During storage at 4 °C, the control group (SDW) exhibited a substantial increase in BI (ΔBI = 0.262), whereas several treatments significantly restricted this progress. Regarding the color stability of the fruit at 4 °C, the D-WKS treatment exhibited the highest resistance to browning with a minimal net increase of only 0.089. This was followed by the D-WKS+Cht combination, which ranked second in suppressing browning by limiting the BI (A_420_) increase to 0.181 throughout the 10-day storage period. Regarding the samples stored at 25 °C, the control group exhibited a significant and rapid progression in enzymatic browning, with a net increase of 0.271 (A_420_) within only 5 days. Among the treatment groups, KL3 demonstrated the highest efficacy in maintaining color stability, showing the lowest ΔBI value of 0.076. This was followed by the GE17+KL3 combination as the second most effective treatment, which limited the BI (A_420_) increase to 0.125. GE17 ranked third with an increase of 0.143 unit, while D-WKS ranked fourth with a ΔBI of 0.146. These findings suggest that at ambient temperatures, the individual and combined applications of these biocontrol agents provide a significant protective effect, effectively buffering the fruit against the rapid acceleration of enzymatic oxidation. A notable disparity was observed in the efficacy of coating materials across different temperatures. At 4 °C, polysaccharide-based coatings, specifically D-WKS and its combination with Cht, provided superior protection. This is likely due to the formation of a robust physical barrier that effectively limits oxygen diffusion and reduces the metabolic rate of the fruit under refrigeration. Conversely, at 25 °C, treatments involving microbial agents (KL3 and GE17) exhibited higher success in limiting the browning rate (ΔBI). This shift can be attributed to the enhanced metabolic activity of the yeasts at ambient temperatures, where they potentially act through biological competition and the induction of host antioxidant defenses. In contrast, the reduced metabolic vigor of these biocontrol agents at 4 °C explains their diminished comparative efficacy in cold storage. Similar to the present study, where a gradual increment in BI values (A_420_) was observed in strawberry fruits throughout the storage period, Aamer et al.^87^ conducted a study on strawberries aimed at maintaining color stability and reported a consistent rising trend in browning intensity over 45 days of storage.

Due to their delicate nature and low physical durability, strawberries are among the most perishable fruits, characterized by a remarkably short shelf life. This fragility is further compounded by a rapid loss of tissue firmness during ripening, which serves as a primary driver of their limited postharvest longevity.^96^ To mitigate these physiological challenges and extend the storage stability of the fruit, various coating materials have been extensively researched. These coatings are thought to act as a semipermeable barrier, potentially regulating gas exchange and moisture loss, which may contribute to maintaining the structural integrity and quality attributes of strawberries throughout the postharvest chain.^97^ Consistent with these protective mechanisms, the application of various coating treatments—comprising polysaccharide-based matrices (chitosan and dextran) and biological control agents (GE17 and KL3)—resulted in significant variations in fruit texture. Storage at 4 °C significantly better-preserved fruit firmness compared to 25 °C (*p* *<* 0.05). At ambient temperature (25 °C), all samples experienced a rapid decline in firmness by Day 5, whereas cold storage maintained higher values even at Day 10 (*p* *<* 0.05). On Day 10, the chitosan and D-WKS+Cht treatments showed the highest firmness levels (7.36 and 7.31 N, respectively). In contrast, the control group dropped to the lowest value (6.20 N). At ambient storage, although all groups softened, the Cht and D-WKS+Cht coatings again outperformed other treatments on Day 5 (7.04 N), effectively slowing down the softening process compared to the control (6.07 N). Regarding the role of biological agents in texture preservation, treatments involving GE17 and KL3 (both alone and in combination) provided better firmness retention than the control but were less effective than chitosan-based coatings in maintaining structural integrity. The results indicate that chitosan-based coatings (Cht and D-WKS+Cht) act as a superior physical barrier, significantly reducing the rate of fruit softening. This protective effect is enhanced when combined with refrigeration (4 °C), which synergistically slows down the metabolic activities and enzymatic degradation of cell walls responsible for tissue softening in strawberries. Similarly, in a study conducted by Huang et al.,^98^ the firmness values of strawberries stored at 10 °C for 12 days exhibited a consistent decline, decreasing from 6 to 4 N. In another study that utilized chitosan as a coating material for strawberries, it was reported that coated samples maintained a final firmness 37.5% higher than the control group after 3 weeks of storage at 4 °C.^5^ This finding further confirms the effectiveness of chitosan as a superior protective matrix for preserving the structural quality of the fruit.

In evaluating storage duration and different coating materials in terms of TPC, it is observed that in both 4 and 25 °C conditions, there is a general decline in TPC levels as the storage period progresses from Day 0 to Day 10. Notably, upon comparing the same storage interval (Day 5), cold storage at 4 °C proves significantly more effective at preserving phenolic compounds than ambient storage at 25 °C (*p* < 0.05). Regarding the percentage loss at 4 °C, the D-WKS + Cht treatment demonstrated the most efficacy in restricting TPC loss to just 7.53%, followed closely by the D-WKS treatment at 7.87%, and the quadruple combination (D-WKS + Cht + GE17 + KL3), which exhibited high stability with a loss of 8.69%. The data clearly demonstrates that while coatings provide a vital barrier, their effectiveness in terms of nutrient retention is maximized when combined with a cold chain (4 °C) to further suppress metabolic TPC depletion, which indicates their superior performance in preserving the fruit’s antioxidant potential. Similar to the results obtained in this study, previous research has also reported a gradual decline in TPC values during the storage of coated strawberries under identical conditions (4 °C and 80% RH).^55^ This decrease is generally attributed to the breakdown of cellular structures during senescence; however, edible coatings may help mitigate this loss, potentially through their effects on moisture loss and oxygen availability, thereby contributing to reduced enzymatic oxidation of phenolic compounds.

#### 3.6.4 Color Evaluation

Color is a primary visual quality attribute that directly influences consumer acceptance and the marketability of strawberries. The changes in color parameters (*L*, *a*, *b*, C*_ab_, *H*°, and Δ*E*) of coated and uncoated strawberries stored under cold (4 °C) and ambient (25 °C) conditions are presented in Table 6. Storage temperature exerted a profound effect on the overall shelf life and color stability of the fruits. Ambient storage (25 °C) severely accelerated the senescence and metabolic decay of the strawberries, restricting the evaluation period to a maximum of 5 days due to rapid microbial spoilage and tissue softening. Conversely, cold storage (4 °C) successfully suppressed the overall respiration rate, enzymatic activities, and moisture loss, allowing the tracking of color characteristics up to 10 days.

**Table 6:** Changes in Color Characteristics of Coated Strawberries under Cold (4 °C) and Ambient (25 °C) Storage Conditions^a^

	experiments	SDW	D-WKS	Cht	D-WKS+Cht	GE17	KL3	GE17 + KL3	D-WKS+Cht+GE17+KL3
**analysis**	**storage days**	**storage at 4 °C**							
*L*	Day 0	37.26 ± 2.42^Aa^	39.46 ± 4.77^Aa^	40.22 ± 2.08^Aa^	40.14 ± 2.67^Aa^	41.56 ± 4.75^Aa^	41.30 ± 3.28^Aa^	38.84 ± 5.25^Aa^	38.33 ± 3.58^Aa^
	Day 5	34.78 ± 1.91^Ba^	38.41 ± 1.94^Aa^	39.00 ± 3.10^Aa^	38.37 ± 2.20^Aa^	39.62 ± 1.47^Aa^	40.31 ± 1.63^ABa^	38.50 ± 4.16^Aa^	36.69 ± 3.31^Aa^
	Day 10	30.13 ± 2.48^Cb^	37.31 ± 2.65^Aa^	35.48 ± 1.26^Aa^	39.65 ± 3.57^Aa^	38.04 ± 0.71^Aa^	35.44 ± 0.69^Ba^	35.05 ± 3.14^Aa^	36.99 ± 0.11^Aa^
*a*	Day 0	39.60 ± 1.73^Aab^	40.43 ± 2.99^Aab^	41.99 ± 1.08^Aa^	40.75 ± 1.49^Aab^	38.38 ± 2.56^Aab^	40.48 ± 1.90^Aab^	40.42 ± 3.98^Aab^	39.87 ± 1.45^Aab^
	Day 5	31.93 ± 6.34^Ad^	39.15 ± 2.70^Aab^	40.98 ± 2.47^Aa^	41.37 ± 2.79^Aa^	38.70 ± 3.28^Aabc^	39.67 ± 2.67^Aab^	38.62 ± 2.99^Aabc^	39.45 ± 2.78^Aab^
	Day 10	39.93 ± 1.36^Aa^	38.93 ± 1.77^Aa^	38.18 ± 2.57^Aa^	39.90 ± 2.56^Aa^	39.60 ± 1.79^Aa^	36.56 ± 5.97^Aa^	32.74 ± 7.94^Aa^	39.83 ± 1.62^Aa^
*b*	Day 0	32.08 ± 7.21^Aab^	35.09 ± 6.17^Aa^	38.02 ± 2.80^Aa^	35.04 ± 3.22^Aa^	37.14 ± 5.88^Aa^	36.80 ± 2.95^Aa^	32.96 ± 10.01^Aab^	32.25 ± 6.10^Aab^
	Day 5	19.88 ± 2.53^Bb^	30.31 ± 5.90^Aab^	31.56 ± 1.99^Bab^	33.15 ± 2.47^Aab^	32.06 ± 3.41^Aab^	32.20 ± 0.55^ABab^	28.70 ± 6.97^Aab^	31.18 ± 4.72^Aab^
	Day 10	30.01 ± 0.64^ABa^	29.97 ± 7.44^Aa^	28.24 ± 1.26^Ba^	33.64 ± 5.41^Aa^	31.16 ± 2.44^Aa^	25.01 ± 4.00^Ba^	25.48 ± 9.83^Aa^	28.50 ± 1.24^Aa^
C*_ab_	Day 0	51.15 ± 5.15^Aab^	53.78 ± 2.55^Aa^	56.66 ± 2.40^Aa^	53.78 ± 2.64^Aa^	53.51 ± 5.10^Aab^	54.78 ± 0.55^Aa^	52.38 ± 8.97^Aab^	51.45 ± 3.67^Aab^
	Day 5	37.64 ± 4.61^Bb^	49.62 ± 5.16^Aab^	51.73 ± 3.01^ABab^	53.01 ± 3.68^Aa^	50.28 ± 4.38^Aab^	51.11 ± 2.18^Aab^	48.27 ± 5.98^Aab^	50.36 ± 4.36^Aab^
	Day 10	49.95 ± 1.16^Aa^	49.36 ± 4.96^Aa^	47.54 ± 1.36^Ba^	52.23 ± 5.42^Aa^	50.40 ± 2.89^Aa^	44.31 ± 7.11^Aa^	41.56 ± 12.25^Aa^	48.99 ± 1.79^Aa^
*H*°	Day 0	38.63 ± 6.17^Aa^	40.81 ± 6.86^Aa^	42.12 ± 1.85^Aa^	40.63 ± 2.49^Aa^	43.87 ± 4.06^Aa^	42.24 ± 3.63^Aa^	38.43 ± 6.91^Aa^	38.72 ± 5.61^Aa^
	Day 5	32.11 ± 2.50^Ab^	37.50 ± 4.64^Aab^	37.59 ± 1.12^ABab^	38.69 ± 0.61^Aab^	39.62 ± 2.08^Aab^	39.11 ± 1.83^ABab^	36.22 ± 5.73^Aab^	38.18 ± 3.77^Aab^
	Day 10	36.94 ± 1.07^Aa^	37.15 ± 7.00^Aa^	36.54 ± 3.03^Ba^	39.95 ± 2.72^Aa^	38.17 ± 1.03^Aa^	34.41 ± 1.40^Ba^	37.12 ± 4.38^Aa^	35.59 ± 1.14^Aa^
Δ*E*	Day 0	75.38 ± 2.43^Aab^	75.29 ± 2.99^Aab^	76.63 ± 1.93^Aa^	74.76 ± 0.91^Aabc^	73.58 ± 1.57^Aabc^	74.54 ± 2.07^Aabc^	75.25 ± 1.54^Aab^	74.67 ± 0.80^Aabc^
	Day 5	69.68 ± 6.26^Aabc^	73.45 ± 1.95^Aabc^	74.27 ± 4.31^Aab^	75.63 ± 1.11^Aa^	72.80 ± 3.92^Aabc^	72.84 ± 2.23^Aabc^	72.99 ± 0.51^Aabc^	75.24 ± 1.57^Aab^
	Day 10	74.59 ± 4.88^Aa^	74.16 ± 1.56^Aa^	74.37 ± 1.29^Aa^	74.20 ± 2.18^Aa^	74.11 ± 2.38Aa	72.72 ± 3.56Aa	71.87 ± 4.43^Aa^	74.11 ± 1.11^Aa^
		**storage at 25 °C**							
*L*	Day 0	38.49 ± 0.87^Aa^	38.21 ± 2.72^Aa^	38.96 ± 3.32^Aa^	39.40 ± 2.51^Aa^	40.39 ± 6.12^Aa^	44.00 ± 4.60^Aa^	35.82 ± 1.84^Ba^	40.27 ± 6.77^Aa^
	Day 5	39.11 ± 2.18^Aa^	40.28 ± 4.89^Aa^	39.55 ± 1.73^Aa^	40.80 ± 1.91^Aa^	41.66 ± 5.45^Aa^	36.84 ± 5.70^Aa^	39.56 ± 2.25^Aa^	42.78 ± 7.62^Aa^
*a*	Day 0	40.19 ± 2.29^Aab^	36.24 ± 3.67^Aab^	38.36 ± 2.02^Aab^	37.81 ± 1.10^Aab^	36.98 ± 1.27^Aab^	36.58 ± 4.78^Aab^	32.31 ± 6.02^Ab^	38.07 ± 0.89^Aab^
	Day 5	37.10 ± 1.38^Aabcd^	35.86 ± 2.22^Aabcd^	37.31 ± 3.52^Aabcd^	37.31 ± 3.25^Aabcd^	39.38 ± 2.63^Aab^	32.38 ± 3.32^Acd^	31.42 ± 6.78^Ad^	33.47 ± 0.02^Bbcd^
*b*	Day 0	31.12 ± 3.33^Aab^	26.76 ± 2.96^Aab^	29.58 ± 6.46^Aab^	27.44 ± 4.35^Aab^	29.59 ± 9.12^Aab^	32.94 ± 4.37^Aab^	22.71 ± 2.87^Aab^	31.71 ± 9.97^Aab^
	Day 5	24.01 ± 3.71^Aab^	26.13 ± 2.63^Aab^	26.11 ± 5.30^Aab^	25.39 ± 4.46^Aab^	34.52 ± 6.90^Aa^	22.41 ± 7.13^Aab^	20.83 ± 3.52^Aab^	20.75 ± 5.28^Aab^
C*_ab_	Day 0	50.92 ± 1.84^Aab^	45.05 ± 4.65^Aab^	48.63 ± 4.42^Aab^	46.79 ± 3.36^Aab^	47.68 ± 6.28^Aab^	49.50 ± 1.24^Aab^	39.51 ± 6.57^Ab^	49.94 ± 6.43^Aab^
	Day 5	44.26 ± 2.53^Aab^	44.38 ± 3.31^Aab^	45.58 ± 5.95^Aab^	45.16 ± 5.16^Aab^	52.52 ± 5.55^Aa^	39.87 ± 1.69^Bab^	37.71 ± 7.49^Ab^	39.56 ± 2.66^Aab^
*H*°	Day 0	37.72 ± 4.04^Aa^	36.42 ± 0.96^Aa^	37.31 ± 6.14^Aa^	35.78 ± 3.72^Aa^	38.01 ± 7.86^Aa^	42.06 ± 7.41^Aa^	35.30 ± 1.75^Aa^	39.02 ± 9.09^Aa^
	Day 5	32.79 ± 4.02^Aab^	36.03 ± 1.19^Aab^	34.74 ± 2.76^Aab^	34.04 ± 2.54^Aab^	40.94 ± 5.20^Aa^	34.39 ± 10.98^Aab^	33.76 ± 2.48^Aab^	31.49 ± 6.75^Ab^
Δ*E*	Day 0	74.13 ± 0.95^Aabc^	70.79 ± 2.58^Aabc^	72.42 ± 0.82^Aabc^	70.92 ± 0.22^Aabc^	70.90 ± 1.54^Babc^	68.96 ± 4.19^Ac^	69.74 ± 1.90^Abc^	72.47 ± 1.62^Aabc^
	Day 5	68.76 ± 0.94^Babc^	68.80 ± 1.74^Aabc^	70.11 ± 3.75^Aabc^	68.92 ± 1.69^Aabc^	73.43 ± 1.98^Aabc^	68.99 ± 4.09^Aabc^	65.38 ± 3.42^Abc^	64.09 ± 5.64^Ac^

a Abbreviations: SDW: Sterile distilled water, D-WKS: Dextran derived from household type grain, Cht: Chitosan, GE17:*Aureobasidium pullulans* GE17, KL3: *Meyerozyma guilliermondii* KL3, uppercase superscripts indicate significant differences among various storage days of the same treatment (with groups at 4 and 25 °C evaluated independently for the identical treatment), significant differences between all of the treatments on the same day are indicated by lowercase superscripts (all trials included in the study, which comprised samples stored at 4 and 25 °C, were analyzed at once).

The *L* value reflects the surface lightness of strawberries, with decreasing values indicating surface darkening and quality deterioration. Over the 10-day cold storage period, a general downward trend was observed across all treatments, signifying a progressive darkening of the fruit; however, the rate of darkening was substantially mitigated by the coating applications. During cold storage at 4 °C, a significant decline (*p* < 0.05) in the L value of the SDW control group was observed, dropping from 37.26 ± 2.42 on Day 0 to 30.13 ± 2.48 on Day 10. This pronounced darkening is directly related to the acceleration of polyphenol oxidase (PPO) activity and surface moisture loss, which trigger rapid oxidative browning in uncoated fruit during senescence. In contrast, all coating formulations, with the exception of the KL3 treatment, successfully maintained the L value compared to the control group, effectively preserving the brightness and visual quality close to that of a fresh strawberry (*p* > 0.05). Among all coated samples, the D-WKS+Cht group was the most successful treatment. It demonstrated the lowest percentage decrease (only 1.22% reduction), with the *L* value changing minimally from 40.14 ± 2.67 on Day 0 to 39.65 ± 3.57 on Day 10. This exceptional color stability highlights the superior efficacy of the D-WKS and chitosan blend in maintaining the fresh appearance of strawberries during cold storage.

The redness (+a) value is considered the most critical color parameter for consumers, as it directly reflects the natural and characteristic appearance of strawberries. This parameter is primarily determined by the fruit’s water-soluble anthocyanin profile, which is dominated by pelargonidin and cyanidin derivatives.^99^ Among these, pelargonidin 3-O-glucoside—the most abundant anthocyanin in cultivated strawberries—imparts the characteristic bright red hue, whereas cyanidin 3-O-glucoside contributes to a darker red pigmentation.^59^ The percentage changes in + *a* values from Day 0 to Day 5 firmly support the protective capacity of the investigated formulations. Under cold storage, the redness (+*a*) value of the uncoated control samples (SDW) experienced a sharp decrease of approximately 19.37% by Day 5, which indicates a significant loss of the characteristic vivid red color of the strawberries due to early tissue deterioration and subsequent enzymatic browning. In contrast, all coated strawberries effectively restricted this color loss to under 4.45% during the same period. Remarkably, the D-WKS+Cht and GE17 treatments induced a slight increase in redness by 1.52 and 0.83%, respectively, demonstrating an active preservation of pigment intensity. This interesting phenomenon is closely linked to the postharvest physiology of the fruit; since strawberries remain metabolically active after harvest, ongoing respiration supports the continuous biosynthesis and accumulation of anthocyanin pigments. While this natural ripening process was masked by oxidative browning in the control group, the biopolymer coatings may have influenced gas exchange and contributed to slowing down senescence. Furthermore, the multicomponent coating (D-WKS+Cht+GE17+KL3) demonstrated near-perfect retention of redness, with a negligible decrease of only 1.05% by the fifth day of cold storage, confirming the synergistic effect of combining structural polysaccharides with antagonistic biocontrol agents in maintaining the visual marketability of the fruits.

According to Dini et al.,^76^ C*_ab_ reflects color saturation or intensity, scaling from the center of the CIELAB color space (C*_ab_ = 0, representing gray) toward pure, vibrant colors (C*_ab_ = 100), where higher values denote greater color purity. When evaluating the C*_ab_ values on Day 5 of cold storage, the control samples (SDW) exhibited the lowest saturation (37.64 ± 4.61), marking a significant decline compared to their initial baseline at Day 0 (*p* < 0.05). Conversely, all coated treatments maintained significantly higher C*_ab_ values than the control group at this storage point (*p* < 0.05). Furthermore, except for the single chitosan coating, none of the other formulation groups showed a statistically significant decrease (*p* > 0.05) in their color saturation levels relative to the beginning of the storage period, demonstrating the efficacy of these treatments in preserving the vividness of the strawberries.

Considering the color parameters collectively, the D-WKS+Cht group emerged as the most effective treatment for preserving the overall color characteristics of strawberries during cold storage, as supported by the stability of the evaluated color parameters (*L*, *a*, *b*, C*_ab_, *H*°, and Δ*E*). In addition to exhibiting exceptional retention of surface lightness (*L*), with only a 1.22% decrease from 40.14 ± 2.67 on Day 0 to 39.65 ± 3.57 on Day 10, this treatment also showed favorable changes in the other chromatic attributes. Specifically, the D-WKS+Cht coating preserved the characteristic redness (a), with a slight increase of 1.52% by Day 5 (41.37 ± 2.79), followed by a value of 39.90 ± 2.56 on Day 10, which remained the highest among the coated treatments. Similarly, the treatment effectively retained the yellow component (b) and exhibited the highest color saturation (C*_ab_) on Day 10 (52.23 ± 5.42), indicating good preservation of the vivid and fresh-like appearance of the fruit. Furthermore, the H° value remained relatively stable throughout storage, while the overall color difference (Δ*E*) showed minimal variation, changing from 74.76 ± 0.91 on Day 0 to 74.20 ± 2.18 on Day 10 (*p* > 0.05).

The relatively large standard deviations observed for some color parameters (e.g., *a*, *b*, and C*_ab_; Table 6) may be attributed to the inherent biological heterogeneity of strawberry fruits and differences in initial pigmentation and ripening stage, despite the use of standardized fruits and separate packages for color analysis. In addition, measurements taken on both sides of each fruit may have captured natural spatial variation in skin color. Such variability is particularly evident for color coordinates that are sensitive to small differences in pigmentation and surface characteristics.

Similarly, Khodaei et al.^55^ evaluated the effects of various edible coatings—including CMC, low methoxyl pectin, Persian gum, and tragacanth gum—on extending the shelf life of fresh strawberries stored at 4 °C for 15 days. In alignment with the findings of the present study, they reported a general reduction in lightness (*L*) over the storage period. Parallel to the results obtained in the current study, they also observed slight fluctuations in the redness (a) value and a gradual decrease in the yellowness (b) value over time, with the coating treatments exhibiting no significant effect on the latter parameter.

#### 3.6.5 Disease Severity (DS) and Infection Ratio (IR)

The 10th day of cold storage (4 °C) revealed vital information about the long-term efficacy of the applied coatings. While the control group (SDW) showed significant spoilage with an IR of 45.45% and a DS of 40%, the experimental treatments using different coating materials demonstrated varying degrees of protection (Figure 4). Notably, the D-WKS treatment provided the most robust defense. This group exhibited the highest level of protection among all tested coatings, achieving the lowest IR (9.09%) and the most suppressed DS (5.45%). The superior performance of D-WKS suggests that the dextran-based EPS layer may act not only as a functional physical barrier limiting oxygen exchange and fungal proliferation, but also potentially through direct or indirect antimicrobial and physiological defense mechanisms that help maintain fruit integrity during cold storage. In addition to the D-WKS treatment, other coating formulations also demonstrated remarkable efficacy by keeping infection and severity levels near or below the 20% threshold after 10 days of cold storage. For instance, the application of GE17 (*A. pullulans*) was exceptionally successful in preventing the onset of decay, matching the lowest recorded IR (9.09%) while maintaining a DS of 20.00%. Similarly, the D-WKS+Cht+GE17+KL3 (quadruple mixture) provided a robust defense by integrating all coating materials and yeasts, which successfully restricted both the occurrence and intensity of the disease with an IR of 18.18% and a DS of 20.00%. Furthermore, the combination of D-WKS+Cht demonstrated superior efficacy by reducing the IR to 18.18% and DS to 21.81%, representing a significant improvement compared to the control group’s much higher values of 45.45 and 40%, respectively. These results indicate that while D-WKS is the superior individual component, the synergy between biocontrol yeasts and the dextran-chitosan matrix offers a highly reliable alternative for extending the shelf life of strawberries under refrigerated conditions. This protection is primarily due to the coating’s ability to act as a semipermeable barrier, which creates a modified internal atmosphere around the fruit. The coating may potentially influence gas exchange, which could contribute to a reduction in the respiration rate of the strawberries.^100^ Furthermore, when combined with refrigeration, this type of suppressed metabolic activity ensures that biochemical changes and tissue degradation occur at a significantly lower rate, thereby maintaining the fruit’s structural integrity against fungal decay.

**Figure 4:**
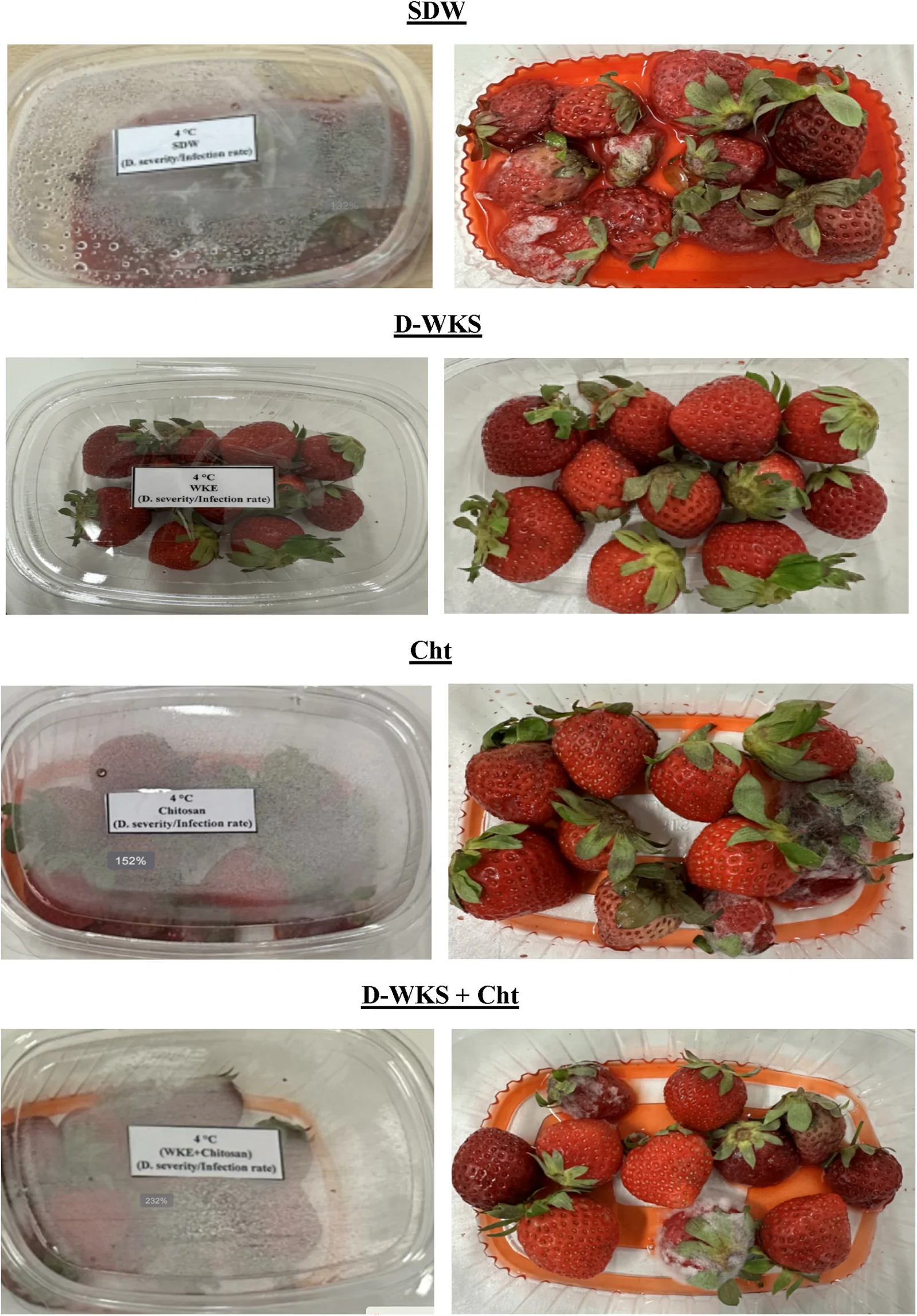

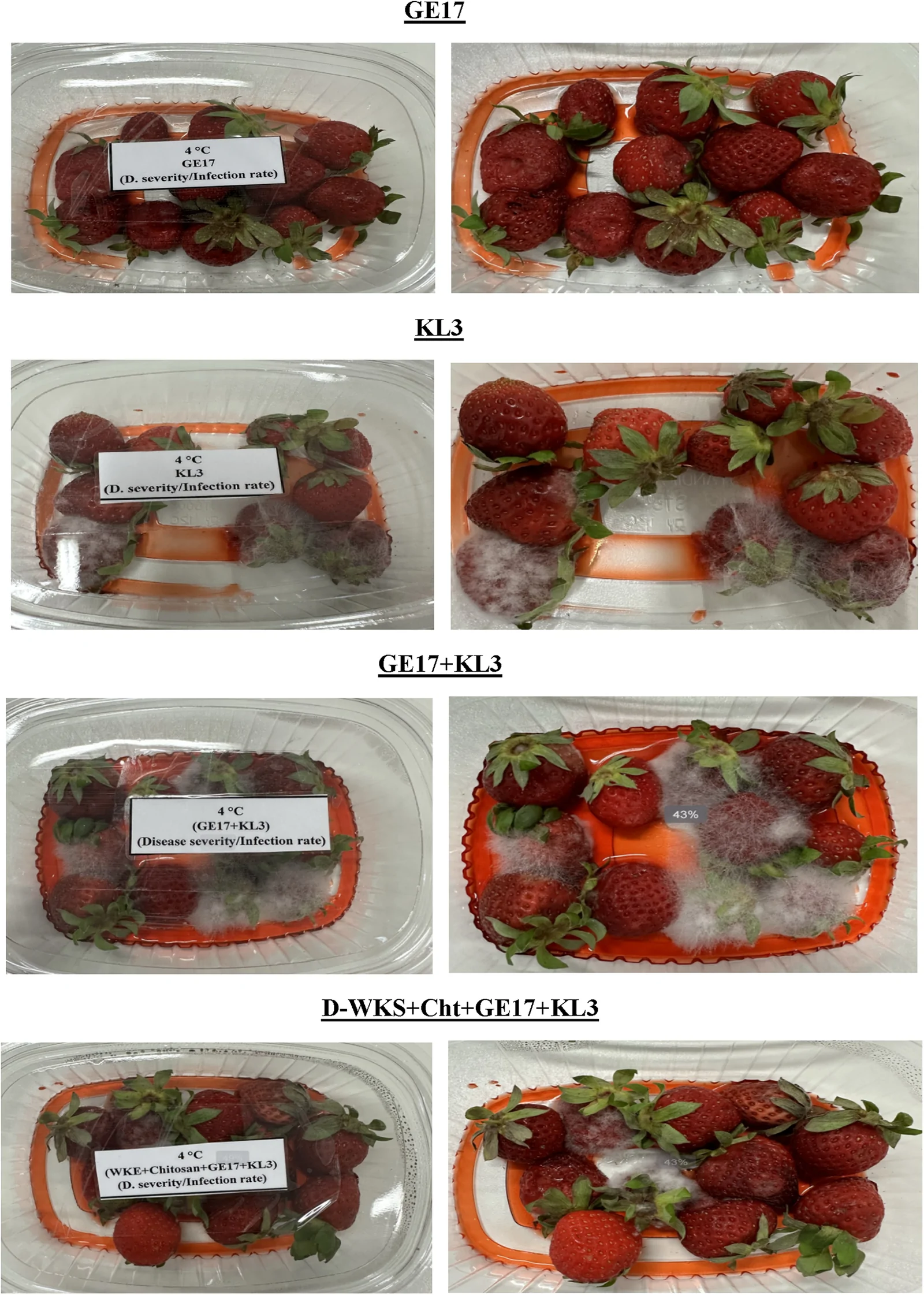
Visual appearance of strawberries subjected to different treatments after 10 days of storage at 4 °C for disease severity (DS) and infection rate (IR) analyses. Abbreviations: D-WKS: Dextran derived from household type grain, Cht: Chitosan, GE17: *Aureobasidium pullulans* GE17, KL3: *Meyerozyma guilliermondii* KL3.

During storage at 25 °C, both the IR and DS showed a drastic increase across all treatments by Day 5. Notably, the effectiveness of D-WKS and Cht, which provided superior protection at 4 °C, declined significantly at this higher temperature; both reached a 100% infection ratio, with D-WKS showing a disease severity of 80% and Cht 74%. In contrast, treatments incorporating biocontrol yeasts demonstrated the highest resilience. KL3 was the most effective, maintaining the lowest values with an IR of 40% and DS of 34%. It was followed by GE17 (IR: 54.54%, DS: 58.18%) and their combination, GE17+KL3 (IR: 60%, DS: 66.66%). These results suggest that while polysaccharide coatings are effective under refrigeration, biological control agents are essential for suppressing fungal growth under ambient temperature stress.

During the storage period, both the IR and DS increased across all treatments. However, the storage temperature had a profound impact on the rate of decay. At 4 °C, strawberries showed significantly better preservation; after 5 days, most treatments (including the control) maintained 0% infection and severity. In contrast, at 25 °C, the decay progressed rapidly, with many groups reaching a 100% infection ratio and high disease severity (up to 80%) by the same day. The stark difference between the two temperatures highlights the critical role of the cold chain. At 25 °C, the high metabolic rate of the fruit and the optimal growth conditions for fungi led to total loss in most groups by Day 5. Conversely, the 4 °C treatment successfully suppressed these biological processes, significantly extending the fruit’s life. This confirms that while the various coatings provide an additional layer of protection, temperature control remains the most vital factor in preventing rapid postharvest spoilage. While IR and DS were quantified macroscopically to reflect practical shelf life extension under natural storage conditions, future integration of quantitative molecular techniques (such as qPCR or high-throughput metabarcoding) will further elucidate the species-specific shifts in the fruit surface mycobiome during storage.

When comparing the current findings with the study by Zhang et al.,^101^ which evaluated the influence of blue light (BL) and salicylic acid (SA) on strawberry quality during 9 days of cold storage, certain differences in efficacy were observed. While the lowest decay incidence reported by Zhang et al.^101^ remained above 20% by day 9, treatments such as D-WKS and GE17 maintained infection ratios below this threshold, even at 10 days of storage. Regarding DS, the data from this study align with the trends observed in the aforementioned literature, confirming that both coating applications and the BL+SA combination are effective strategies for limiting decay intensity and supporting the postharvest life of strawberries.

#### 3.6.6 Sensory Evaluation of Coated Strawberries during Cold and Ambient Storage

Principal component analysis (PCA) was performed to determine the effects of different coating materials, storage temperatures, and storage times on the sensory attributes of strawberries (Figure 5A). A high percentage of the total variance (90.76%) was explained by the first two principal components, where the first component (F1) comprised 80.84% and the second component (F2) constituted 9.93%. F1 is the dominant axis, that primarily separates the samples based on storage temperature (4 vs 25 °C) and storage duration (Day 5 vs Day 10). Moving from left to right along the F1 axis represents a transition from deteriorated sensory characteristics to fresher, high-quality attributes. High quality attributes (overall quality, color, taste, firmness, and acidity) are clustered closely together on the far-right side of the F1 axis. This indicates that these positive sensory traits are highly correlated with each other. According to the correlation matrix (Supporting Table 2a), overall quality exhibited extremely strong positive correlations with primary freshness indicators: taste (*r* = 0.969), firmness (*r* = 0.932), color (*r* = 0.908). Concurrently, a highly strong and significant positive correlation was observed between the two negative sensory traits, shrivelling and off-flavor (*r* = 0.968), indicating that physiological tissue degradation and water loss during storage are concurrently accompanied by the development of undesirable flavor compounds. Interestingly, while sweetness appears visually separated along the F2 axis on the biplot, the correlation matrix reveals it still maintains moderate-to-strong positive associations with Shrivelling (*r* = 0.730) and Off-flavor (*r* = 0.706). This correlation suggests that as strawberries undergo senescence, moisture loss concentrated the soluble solids (perceived sweetness), even while unacceptable spoilage characteristics developed. On the other hand, F2 axis explains a much smaller portion of the variance and is mostly related to specific taste nuances like sweetness (positive F2) versus astringency (negative F2).

**Figure 5:**
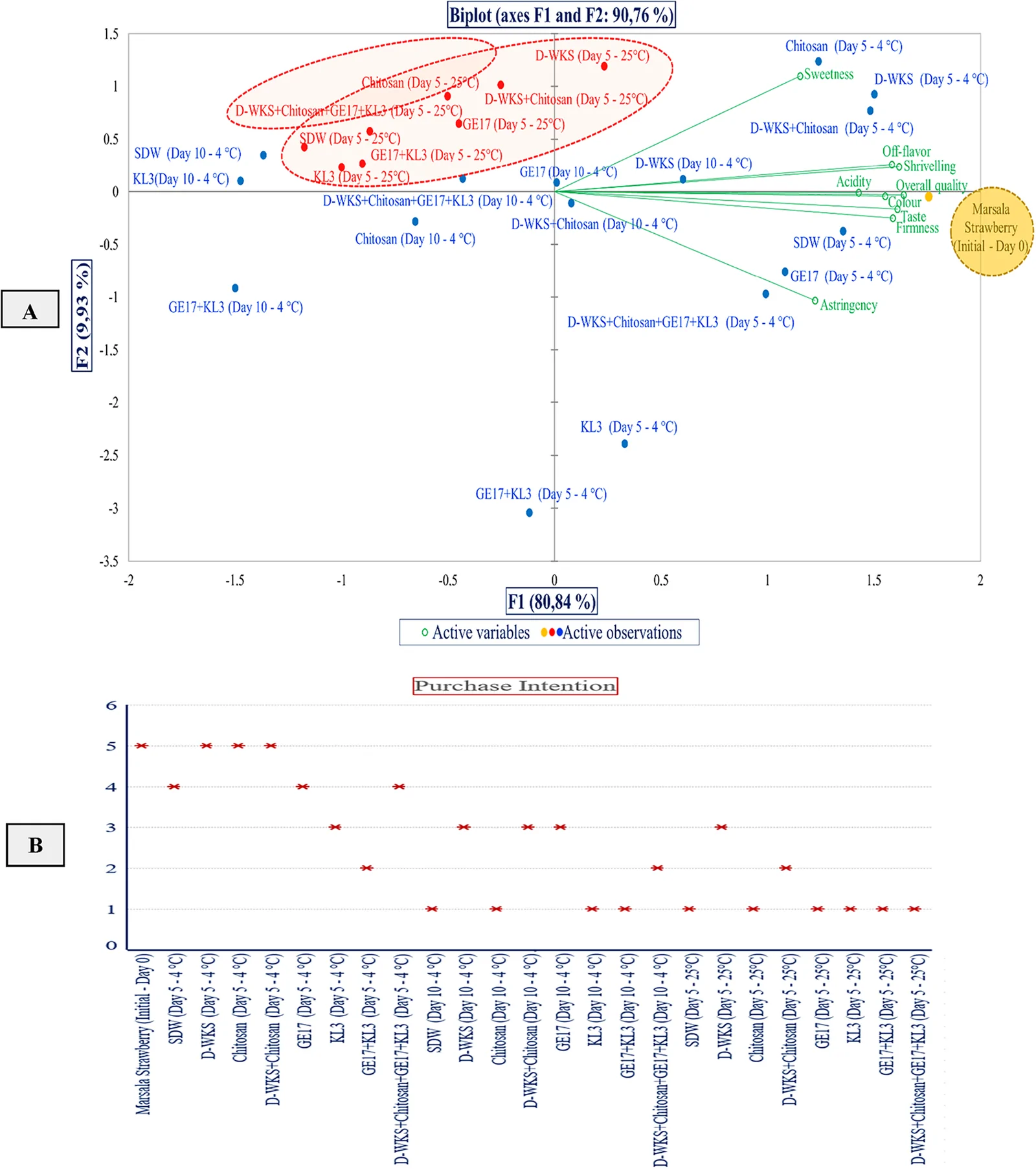
(A) PCA biplot illustrating the evolution of sensory profiles in strawberries as affected by different coating treatments and storage conditions (4 and 25 °C) over time. Variables are shown in green (text and open markers), while observations are color-coded by storage condition: initial prestorage strawberries in yellow, 4 °C storage trials in blue, and 25 °C storage trials in red (matching text and filled markers), (B) Changes in panelists’ purchase intention scores of strawberry samples coated with various formulations during storage at 4 and 25 °C as a function of storage time.

Additional details regarding the PCA model, including descriptive statistics of the quality attributes, eigenvalues with variance distribution, factor loading matrices, and variable contributions (%) to the principal components (F1–F9) are summarized in Supporting Table 2b–e.

Regarding storage temperature, most of the strawberry samples stored at ambient temperature (25 °C for 5 days, represented by red dots) clustered heavily within the negative F1 region (upper-left quadrant), moving drastically away from the “Marsala Strawberry (Initial - Day 0)” benchmark. These samples were negatively correlated with overall quality, taste, firmness, color, and acidity. This clear shift demonstrates that storing strawberries at room temperature (25 °C), even for just 5 days, triggers a rapid and unmitigated decline in marketability, physical integrity, and sensory acceptance regardless of the coating used. However, a highly noteworthy exception was observed for D-WKS (Day 5–25 °C), which separated itself from the rest of the ambient storage group and positioned uniquely in the upper-right quadrant. While still suffering from some storage stress, the solo D-WKS coating demonstrated a remarkable ability to resist total sensory collapse even at 25 °C, maintaining a closer proximity to the freshness indicators than any other treatment at room temperature. This distinctive performance highlights the superior protective barrier property of the D-WKS coating, making it a highly promising candidate for preserving strawberry quality under suboptimal thermal conditions.

In contrast, refrigeration at 4 °C effectively arrested this rapid decay. At Day 5, the samples coated with Chitosan (Day 5–4 °C), D-WKS (Day 5–4 °C) and their combination (D-WKS+Chitosan Day 5–4 °C) were positioned closest to the positive vectors of overall quality, color, taste, and firmness, clustering tightly near the baseline fresh control (Marsala strawberry initial - day 0). This positioning indicates excellent preservation of physical and organoleptic properties during short-term cold storage. As cold storage progressed to Day 10, a distinct leftward shift along the F1 axis occurred, highlighting the expected sensory decline over time; however, strawberries coated with D-WKS (Day 10–4 °C), D-WKS+Chitosan (Day 10–4 °C), and GE17 (Day 10–4 °C) remained significantly closer to the center of the plot, demonstrating a delayed degradation process. The negative control SDW (Day 10–4 °C) and treatments like KL3 (Day 10–4 °C) shifted furthest to the left, indicating severe quality loss and an increase in negative traits like shrivelling and off-flavor.

In conclusion, temperature management is the most critical parameter for maintaining strawberry shelf life, as ambient storage (25 °C) leads to rapid sensory failure. Under proper cold storage (4 °C), standalone treatments of Chitosan, D-WKS and their combination provide the best sensory preservation up to Day 5. For extended storage (Day 10), formulation coatings incorporating D-WKS or D-WKS+Chitosan demonstrated the highest efficacy in delaying senescence, minimizing shrivelling, and depressing off-flavor development compared to uncoated negative controls. Specifically, the D-WKS (4 °C) treatment emerged as by far the most successful standalone application in preserving strawberry quality. Notably, it was the only 10-day sample that managed to remain on the positive side of the F1 axis, further demonstrating its superior efficacy in maintaining overall quality during extended storage.

Parallel to the sensory profile evolution observed in the PCA biplot, the consumers’ purchase intention scores further validated the critical impacts of storage temperature, duration, and coating formulations (Figure 5B). Initially (Day 0), the fresh control sample (“Marsala Strawberry”) received the maximum purchase intention score of 5, reflecting its pristine quality parameters. Under refrigerated conditions (4 °C), the decline in purchase intention was significantly suppressed, especially during short-term storage. Most notably, at Day 5 of cold storage, the strawberry samples coated with D-WKS, Chitosan, and the D-WKS+Chitosan combination achieved the maximum purchase intention score of 5. It is highly remarkable that these three treatments received identical scores to the prestorage fresh control (Day 0) from the panelists, indicating that they retained nearly the same sensory attributes and marketability as the fresh strawberries.

As cold storage extended to Day 10, a generalized reduction in purchase intention scores occurred due to natural senescence. Nevertheless, distinct formulation-dependent differences were clear. The negative control SDW (Day 10–4 °C) and treatments like KL3, Chitosan, and GE17+KL3 dropped to the minimum score of 1, showing a total loss of marketability due to advanced shrivelling and off-flavors. Particularly, while the standalone Chitosan treatment demonstrated high efficacy in maintaining premium quality up to Day 5, it failed to extend this protective effect to Day 10 when applied alone, shifting heavily into the negative F1 region (lower-left quadrant) of the PCA biplot (Figure 5A). However, when Chitosan was combined with D-WKS (D-WKS+Chitosan), a remarkable synergistic effect was observed, effectively preventing this negative shift and allowing the combination to successfully maintain consumer acceptance at Day 10. In addition to the D-WKS+Chitosan formulation, the strawberries coated with D-WKS (Day 10–4 °C) and GE17 (Day 10–4 °C) also effectively counteracted this degradation, sustaining the highest purchase intention score of 3 among all 10-day samples. This finding perfectly aligns with the PCA biplot, where these three specific groups remained significantly closer to the center, demonstrating a prolonged commercial shelf life. Regarding ambient storage (25 °C), a dramatic decline in purchase intention was observed across nearly all treatments by Day 5. Reflecting their unfavorable positioning within the negative F1 region of the PCA biplot, the negative control (SDW) and most coating formulations dropped straight to the lowest score of 1, indicating a total loss of marketability and sensory rejection. However, in perfect agreement with the PCA trends, the standalone D-WKS (Day 5–25 °C) treatment stood out as a remarkable exception by maintaining a moderate purchase intention score of 3. This distinct performance highlights the superior ability of the D-WKS coating to act as a robust protective barrier, delaying rapid sensory deterioration and retaining commercial acceptability even under suboptimal, high-temperature conditions.

While the sensory evaluation in the present study was conducted with a panel of seven trained evaluators, the panel size was constrained by the demanding experimental matrix involving 25 distinct sample groups across storage periods and the limited availability of nondecayed fruit at each interval. The use of the same trained panelists throughout the study helped reduce interindividual variability and maintain scoring consistency across assessment periods. Nevertheless, a panel size of seven represents a limitation in terms of statistical power and may limit the generalizability of the sensory findings. Future consumer-oriented studies involving a larger number of untrained panelists could provide further insights into broader consumer acceptance and marketability.

Despite the promising results obtained in this study, some limitations should be acknowledged. First, molecular-level characterization of the dextran, particularly its molecular weight and molecular weight distribution, was not performed, which limits a more detailed understanding of the relationship between dextran structure and its functional properties. Second, the permeability properties of the resulting coatings, including water vapor and gas permeability, were not characterized. Such measurements could provide further insight into the barrier properties of the coatings and help explain their effects on moisture loss, respiration, and other quality attributes of strawberries during storage. In addition, the spoilage microorganisms associated with the strawberry surface were not identified at the species level, limiting a more detailed assessment of the microbial changes underlying the observed shelf life effects. Future studies should therefore incorporate molecular characterization of dextran, systematic evaluation of coating permeability, and species-level characterization of spoilage microorganisms to better elucidate the structure–function relationships and microbial mechanisms underlying the performance of the coatings.

## 4 Conclusions

This study demonstrates the successful extraction and comparative evaluation of two distinct water kefir grains—industrial and household types—and details the structural and functional characterization of their derived dextrans. The household grain dextran (D-WKS) exhibited superior in vitro antioxidant and broad-spectrum antifungal activities compared to its industrial counterpart, highlighting its potential as a promising biomacromolecule for food preservation. Moving beyond in vitro characterization, this research comprehensively evaluated the polysaccharide dextran as an eco-friendly edible coating to extend the postharvest shelf life of strawberries, testing it not only as a standalone application but also in direct combination with chitosan, two antagonistic yeast species (*A. pullulans* GE17 and *M. guilliermondii* KL3), and their multicomponent formulations.

The storage trials highlighted that while refrigeration remains important for delaying fruit senescence, the application of coatings based on these natural polysaccharides (dextran and chitosan) can enhance quality retention under the storage conditions evaluated. When applied as standalone treatments, both biopolymers may act as semipermeable barriers, potentially influencing gas exchange and moisture loss up to Day 5. However, when storage was extended to 10 days, the efficacy of the individual chitosan treatment decreased, whereas the standalone D-WKS application continued to exhibit a moderate protective effect. Ultimately, the results indicate that combining chitosan with the D-WKS matrix may be important for maintaining the effectiveness of chitosan during extended storage, with the D-WKS–chitosan combination providing better preservation of several quality attributes and consumer acceptability than the individual chitosan treatment.

As a future perspective, further investigations should focus on elucidating the molecular interactions within the D-WKS and chitosan blend and their relationship with coating properties. Such studies could contribute to the further optimization and development of these coatings for larger-scale applications. Additionally, broader sensory evaluation involving a wider consumer panel, together with techno-economic and environmental assessments, would be valuable for further evaluating the potential practical and commercial applicability of water kefir-derived polysaccharides in postharvest preservation.

## Supplementary Material



## Data Availability

Data will be made available upon request.

## Notes

Results of titratable acidity and statistical analyses under ambient and cold storage conditions (Supp.1); Pearson correlation matrix between the evaluated quality variables (Supp. 2a); eigenvalues and total variance explained by principal components (F1–F9) (Supp. 2b); factor loading matrix of quality attributes on principal components (F1–F9) (Supp. 2c); descriptive statistics of quantitative quality parameters (Supp. 2d); and contribution (%) of the variables to the principal components (F1–F9) derived from PCA (Supp. 2e) (PDF)
