# Supplementary material for: Characterization and Application of Water Kefir-Derived Dextran as Coatings with Chitosan and Antagonistic Yeasts for Strawberry Preservation
Source: ACS Omega. 2026 Oct 14;11(42):65840–64. doi: 10.1021/acsomega.6c08930 (PMC13717806; doi:10.1021/acsomega.6c08930)
Supplement: Supplementary file 1 [file ao6c08930_si_001.pdf]

Title: Characterization and Application of Water Kefir-Derived Dextran as Coatings with Chitosan and Antagonistic Yeasts for Strawberry Preservation

Authorship: Bilal Agirman*

Affiliation: Cukurova University, Engineering Faculty, Department of Food Engineering, 01330, Adana, Türkiye

E-mail address: bagirman@cu.edu.tr (corresponding author)

Supp.1. Results of titratable acidity and statistical analyses under ambient and cold storage conditions

		4 °C			25 °C	
	Treatments	Day 0	Day 5	Day 10	Day 0	Day 5
TA (%)*	SDW	0.64 ± 0.0007^{Ca}	0.79 ± 0.0001^{An}	0.67 ± 0.0001^{Bh}	0.64 ± 0.0005^{Ba}	1.58 ± 0.0004^{Ab}
	D-WKS	0.56 ± 0.0007^{Cd}	0.87 ± 0.0002^{Bj}	0.91 ± 0.0001^{Ac}	0.56 ± 0.0003^{Bd}	1.33 ± 0.0002^{Ae}
	Cht	0.50 ± 0.0001^{Ck}	0.87 ± 0.0001^{Bi}	0.94 ± 0.0002^{Ab}	0.50 ± 0.0003^{Bk}	1.37 ± 0.0002^{Ac}
	D-WKS+Cht	0.54 ± 0.0002^{Cf}	0.79 ± 0.0000^{Bm}	0.97 ± 0.0000^{Aa}	0.54 ± 0.0003^{Be}	1.31 ± 0.0004^{Af}
	GE17	0.52 ± 0.0002^{Ci}	0.80 ± 0.0003^{Bl}	0.86 ± 0.0001^{Ae}	0.52 ± 0.0001^{Bj}	1.28 ± 0.0004^{Ag}
	KL3	0.59 ± 0.0001^{Cb}	0.77 ± 0.0002^{Bo}	0.87 ± 0.0002^{Ad}	0.59 ± 0.0002^{Bc}	1.34 ± 0.0002^{Ad}
	GE17+KL3	0.53 ± 0.0002^{Ch}	0.77 ± 0.0002^{Bp}	0.85 ± 0.0001^{Af}	0.53 ± 0.0002^{Bh}	1.25 ± 0.0004^{Ah}
	D-WKS+Cht+GE17+KL3	0.54 ± 0.0004^{Cg}	0.83 ± 0.0003^{Ak}	0.80 ± 0.0005^{Bg}	0.54 ± 0.0002^{Bg}	1.72 ± 0.0007^{Aa}

Abbrevations: *: as citric acid, SDW: Sterile distilled water, D-WKS: Dextran derived from household type grain, Cht: Chitosan, GE17: *Aureobasidium pullulans* GE17, KL3: *Meyerozyma guilliermondii* KL3, uppercase superscripts indicate significant differences among various storage days within the same treatment (with groups stored at 4 °C and 25 °C evaluated independently for each treatment), lowercase superscripts indicate significant differences among all treatments on the same storage day (where all trials comprising samples stored at 4 °C and 25 °C were analyzed simultaneously).

Supp 2. (a) Pearson correlation matrix between the evaluated quality variables

Variables	Colour	Firmness	Sweetness	Acidity	Astringency	Shrivelling	Off-flavor	Taste	Overall quality
Colour	**1**	0,892	0,620	0,748	0,681	0,883	0,863	0,886	0,908
Firmness	0,892	**1**	0,554	0,794	0,763	0,900	0,866	0,946	0,932
Sweetness	0,620	0,554	**1**	0,604	0,178	0,730	0,706	0,596	0,640
Acidity	0,748	0,794	0,604	**1**	0,653	0,795	0,787	0,738	0,810
Astringency	0,681	0,763	0,178	0,653	**1**	0,599	0,571	0,760	0,711
Shrivelling	0,883	0,900	0,730	0,795	0,599	**1**	0,968	0,944	0,971
Off-flavor	0,863	0,866	0,706	0,787	0,571	0,968	**1**	0,910	0,949
Taste	0,886	0,946	0,596	0,738	0,760	0,944	0,910	**1**	0,969
Overall quality	0,908	0,932	0,640	0,810	0,711	0,971	0,949	0,969	**1**

(b) Eigenvalues and total variance explained by principal components (F1–F9)

	F1	F2	F3	F4	F5	F6	F7	F8	F9
Eigenvalue	7,275	0,893	0,339	0,195	0,144	0,089	0,036	0,016	0,013
Variability (%)	80,837	9,927	3,765	2,170	1,600	0,984	0,397	0,173	0,148
Cumulative %	80,837	90,764	94,528	96,699	98,298	99,283	99,679	99,852	100,000

(c) Factor loading matrix of quality attributes on principal components (F1–F9)

	F1	F2	F3	F4	F5	F6	F7	F8	F9
Colour	0,932	-0,026	-0,099	0,117	-0,321	-0,066	-0,009	0,006	0,012
Firmness	0,953	-0,151	-0,055	0,001	-0,035	0,245	0,063	-0,002	-0,024
Sweetness	0,691	0,657	0,120	0,262	0,078	0,008	0,013	-0,008	-0,007
Acidity	0,857	-0,005	0,487	-0,153	-0,049	0,015	-0,026	-0,002	0,023
Astringency	0,733	-0,619	0,124	0,206	0,110	-0,088	0,031	0,014	-0,018
Shrivelling	0,971	0,143	-0,102	-0,100	0,057	-0,020	-0,052	0,097	-0,028
Off-flavor	0,950	0,155	-0,097	-0,176	0,042	-0,115	0,131	-0,017	0,009
Taste	0,966	-0,100	-0,175	0,026	0,113	0,038	-0,052	-0,013	0,087
Overall quality	0,984	-0,019	-0,089	-0,073	0,036	-0,035	-0,086	-0,074	-0,056

(d) Descriptive statistics of quantitative quality parameters

Variable	Observations	Obs. with missing data	Obs. without missing data	Minimum	Maximum	Mean	Std. deviation
Colour	25	0	25	1,300	8,500	5,548	2,101
Firmness	25	0	25	1,500	8,700	4,563	2,609
Sweetness	25	0	25	4,000	8,200	6,639	1,064
Acidity	25	0	25	3,500	8,000	6,369	1,096
Astringency	25	0	25	6,300	9,000	7,612	0,940
Shrivelling	25	0	25	1,000	9,000	4,292	2,622
Off-flavor	25	0	25	1,000	9,000	4,848	2,645
Taste	25	0	25	1,000	8,500	4,384	2,622
Overall quality	25	0	25	1,000	9,000	4,564	2,536

(e) Contribution (%) of the variables to the principal components (F1–F9) derived from PCA

	F1	F2	F3	F4	F5	F6	F7	F8	F9
Colour	11,931	0,075	2,890	7,042	71,675	4,882	0,229	0,198	1,078
Firmness	12,489	2,539	0,886	0,000	0,833	67,729	11,023	0,015	4,487
Sweetness	6,572	48,364	4,274	35,236	4,223	0,064	0,504	0,437	0,326
Acidity	10,104	0,003	70,132	11,994	1,654	0,257	1,834	0,019	4,003
Astringency	7,395	42,876	4,569	21,637	8,344	8,655	2,724	1,254	2,547
Shrivelling	12,956	2,297	3,078	5,071	2,274	0,456	7,659	60,272	5,937
Off-flavor	12,414	2,694	2,749	15,904	1,228	14,927	47,724	1,784	0,577
Taste	12,836	1,111	9,081	0,352	8,891	1,656	7,557	1,066	57,451
Overall quality	13,303	0,040	2,342	2,764	0,879	1,375	20,747	34,955	23,594
